# High-yield, wafer-scale fabrication of ultralow-loss, dispersion-engineered silicon nitride photonic circuits

**DOI:** 10.1038/s41467-021-21973-z

**Published:** 2021-04-16

**Authors:** Junqiu Liu, Guanhao Huang, Rui Ning Wang, Jijun He, Arslan S. Raja, Tianyi Liu, Nils J. Engelsen, Tobias J. Kippenberg

**Affiliations:** Institute of Physics, Swiss Federal Institute of Technology Lausanne (EPFL), Lausanne, Switzerland

**Keywords:** Integrated optics, Silicon photonics, Nonlinear optics

## Abstract

Low-loss photonic integrated circuits and microresonators have enabled a wide range of applications, such as narrow-linewidth lasers and chip-scale frequency combs. To translate these into a widespread technology, attaining ultralow optical losses with established foundry manufacturing is critical. Recent advances in integrated Si_3_N_4_ photonics have shown that ultralow-loss, dispersion-engineered microresonators with quality factors *Q* > 10 × 10^6^ can be attained at die-level throughput. Yet, current fabrication techniques do not have sufficiently high yield and performance for existing and emerging applications, such as integrated travelling-wave parametric amplifiers that require meter-long photonic circuits. Here we demonstrate a fabrication technology that meets all requirements on wafer-level yield, performance and length scale. Photonic microresonators with a mean *Q* factor exceeding 30 × 10^6^, corresponding to 1.0 dB m^−1^ optical loss, are obtained over full 4-inch wafers, as determined from a statistical analysis of tens of thousands of optical resonances, and confirmed via cavity ringdown with 19 ns photon storage time. The process operates over large areas with high yield, enabling 1-meter-long spiral waveguides with 2.4 dB m^−1^ loss in dies of only 5 × 5 mm^2^ size. Using a response measurement self-calibrated via the Kerr nonlinearity, we reveal that the intrinsic absorption-limited *Q* factor of our Si_3_N_4_ microresonators can exceed 2 × 10^8^. This absorption loss is sufficiently low such that the Kerr nonlinearity dominates the microresonator’s response even in the audio frequency band. Transferring this Si_3_N_4_ technology to commercial foundries can significantly improve the performance and capabilities of integrated photonics.

## Abstract (web summary)

For widespread technological application of nonlinear photonic integrated circuits, ultralow optical losses and high fabrication throughput are required. Here, the authors present a CMOS fabrication technique that realizes integrate photonic microresonators on waver-level with mean quality factors exceeding 30 million and 1 dB/m optical losses.

## Introduction

Silicon photonics^1,2^ has evolved into a mature technology enabling the generation, modulation, and detection of optical signals on-chip, via heterogeneous or hybrid integration of different materials^3–5^. Many integrated devices have been demonstrated using silicon photonics, including silicon-based lasers^6,7^. Within the past two decades, they have been transferred from academic research to large-volume commercial deployment in datacenter interconnects. A second revolution is currently under way, where the optical nonlinearities of photonic integrated circuits (PICs)—accessed with continuous-wave lasers at sub-milliwatt power—can enable novel devices, of which Kerr frequency combs have been one of the studied processes. Dissipative Kerr soliton frequency combs (“soliton microcombs”)^8,9^ constitute chip-scale frequency combs of broad bandwidths and repetition rates in the terahertz to microwave domain, and offer a route to highly compact frequency combs for a variety of applications in airborne systems or space. Microcombs are compatible with wafer-scale manufacturing as well as hybrid integration with III-V/Si lasers^10–12^, and have already been used in various system-level demonstrations including coherent telecommunications^13,14^, astronomical spectrometer calibration^15,16^, ultrafast ranging^17,18^, massively parallel coherent LiDAR^19^, frequency synthesizers^20^, atomic clock architectures^21^, and neuromorphic computing^22,23^. Many integrated nonlinear photonic platforms for microcombs have emerged, ranging from Si_3_N_4_^24–30^, diamond^31^, tantala^32^, and SiC^33^ to highly nonlinear AlGaAs^34,35^ and GaP^36^ on insulator, as well as electro-optic platforms such as LiNbO_3_^37–40^ and AlN^41–43^.

**Fig. 1:**
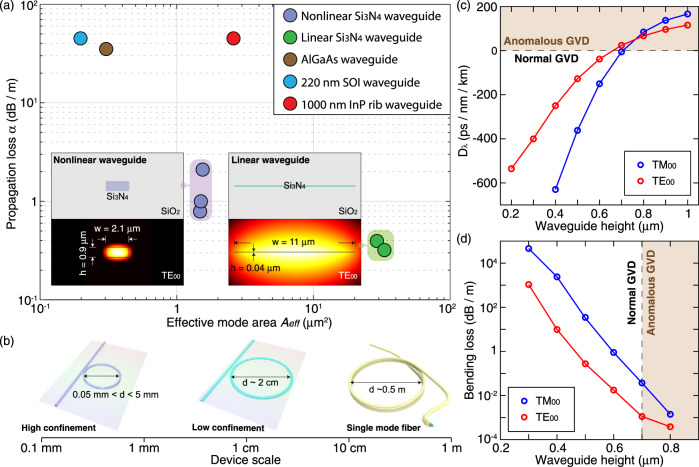
Comparison of ultralow-loss linear and nonlinear Si_3_N_4_ platforms with state-of-the-art silicon, InP and AlGaAs platforms. **a** Comparison of optical losses and effective mode areas (in the telecommunication band of 1550 nm) in state-of-the-art, low-loss waveguides, including nonlinear (i.e. thick-core, refs. ^24,25^ and this work) and linear (i.e. thin-core, refs. ^49,50^) Si_3_N_4_ waveguides, 220 nm silicon-on-insulator (SOI) waveguides^54^, 1000 nm InP rib waveguides^55^, and nonlinear AlGaAs waveguides^35^. The insets show the waveguide geometry and optical mode profile of the Si_3_N_4_ waveguides. **b** Comparison in device size for linear and nonlinear Si_3_N_4_ waveguides and single-mode fibers. **c** Simulated GVD parameter *D*_*λ*_ as a function of the waveguide height *h*, with a fixed waveguide width of *w* = 2.1 *μ*m. **d** Simulated bending loss as a function of the waveguide height *h*, with a fixed bending radius of *d*/2 = 25 *μ*m and waveguide width of *w* = 2.1 *μ*m. Anomalous-GVD region is brown-shaded, which is accessed with *h* > 700 nm. The wavelength studied here is 1550 nm.

For integrated nonlinear photonics, in particular soliton microcombs, Si_3_N_4_^44^ has emerged as a leading material due to its ultralow optical losses, absence of two-photon absorption in the telecommunication bands, strong Kerr nonlinearity, high refractive index, space compatibility^45^ and exceptionally high power handling capability^46,47^. To date, among all integrated photonic platforms^48^, optical losses near or below 1 dB m^−1^ have only been demonstrated in Si_3_N_4_ PICs. While thin-core waveguides (i.e. waveguide height *h* < 100 nm)^49–51^ have achieved ultralow losses, they are not suitable for nonlinear photonics due to the low effective refractive indices, low Kerr nonlinearity and large mode areas. Only recently, nonlinear, thick-core waveguides (i.e. *h* > 700 nm)^24–26^ enabling negligible bending losses, dispersion engineering, and high Kerr nonlinearity, have been demonstrated, as outlined in Fig. 1. Most of the aforementioned system-level demonstrations of soliton microcombs^13,15,17,19–21^ are based on this type of Si_3_N_4_ PICs. In addition, power-efficient supercontinuum generation has also been attained in the near-infrared as well as mid-infrared, enabling dual-comb spectrscopy^52,53^. Figure 1a highlights the lowest-loss thick-core (refs. ^24,25^ and this work) and thin-core (refs. ^49,50^) Si_3_N_4_ waveguides in terms of their optical losses and effective areas of the fundamental optical mode, in comparison with the state-of-the-art, lowest-loss silicon^54^, InP^55^, and AlGaAs^35^ waveguides. The tight confinement significantly reduces the bending loss, a key parameter for device footprint and photonic integration, as outlined in Fig. 1b. Though the desirable combination of tight confinement, ultralow loss, and engineered anomalous group-velocity dispersion (GVD) has been achieved^24,25^, it has only been attained in individual chips, i.e. with die-level throughput. Meanwhile, the fabrication of densely packed, meter-long PIC has not yet been realized, and neither has wafer-level fabrication yield, reliability and reproducibility, required for widespread adoption in CMOS foundries. Yet, densely packed, meter-long nonlinear Si_3_N_4_ PIC could enable a new class of devices, ranging from integrated traveling-wave parametric amplifiers^56–59^ to integrated mode-locked-lasers based on rare-earth doping^60^.

Here we report a high-yield, wafer-scale fabrication technology to build tight confinement, ultralow-loss, dispersion-engineered Si_3_N_4_ waveguides of length exceeding one meter. This Si_3_N_4_ fabrication technology is based on the photonic Damascene process^61^ using standard CMOS fabrication techniques such as DUV stepper lithography, dry etching, and low-pressure chemical vapor deposition (LPCVD). Integrated Si_3_N_4_ microresonators fabricated using this process are systematically characterized and analyzed, showing quality (*Q*) factors above 30 × 10^6^, linear losses of 1 dB m^−1^, and wafer-level yield.

## Results

### Wafer-scale-yield photonic Damascene process

**Fig. 2:**
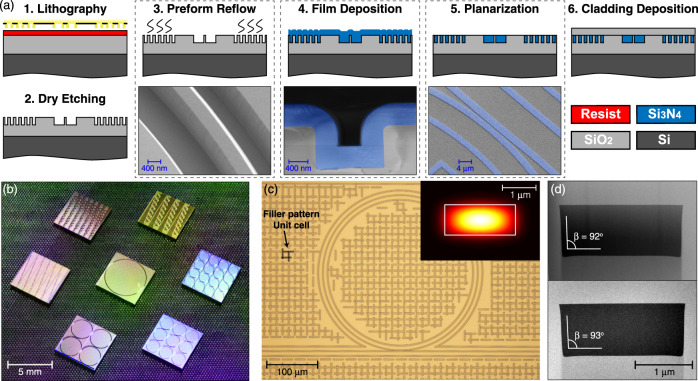
The wafer-scale photonic Damascene process flow and highlighted features. **a** Process flow of the optimized photonic Damascene process including DUV stepper lithography, preform dry etching, preform reflow, LPCVD Si_3_N_4_ deposition, planarization, and SiO_2_ cladding deposition. The blue shaded parts are Si_3_N_4_. **b** Photograph showing Si_3_N_4_ photonic chips with microring resonators of different FSRs. **c** Optical micrograph showing the bus waveguide, microring resonator, and filler patterns (used to prevent crack formation). Inset: simulated tightly confined optical mode. **d** Transmission electron micrographs (TEM) of the waveguide cross-sections, before (top) and after (bottom) the preform reflow. The reflow preserves the waveguide dimensions accurately, while removing high-frequency spatial roughness.

Figure 2a shows the process flow of the optimized photonic Damascene process, and scanning electron micrographs (SEM) for selected key steps. The waveguides and stress-release filler patterns^61,62^ are written directly on the SiO_2_ substrate via DUV stepper lithography based on a 248 nm KrF excimer laser. The use of DUV, in contrast to the commonly employed electron-beam lithography, enables a dramatic increase in fabrication throughput, stability, and reproducibility, essential to large-volume manufacturing. The patterns are then dry-etched into the SiO_2_ substrate to create waveguide preforms. We note that our SiO_2_ dry etching does not introduce a trade-off between the etch verticality and surface roughness. Figure 2d top shows the sidewall bottom angle 90^∘^ < *β* < 92^∘^. To further reduce the waveguide’s root mean square sidewall roughness to sub-nanometer level, the substrate is annealed at 1250 ^∘^C (“preform reflow”)^63^. Importantly, this reflow process can further reduce the scattering loss (see Supplementary Information), and does not lead to significant deformation of the waveguide preform. Figure 2d bottom shows the measured sidewall bottom angle *β* ≈ 93^∘^, after reflow. An LPCVD Si_3_N_4_ film of 1000 nm thickness is deposited on the patterned substrate, filling the preform trenches and forming the waveguides. To improve the yield and allow wafer-scale processing, an etchback planarization process is applied (see Supplementary Information), combining photoresist coating, dry etching and chemical–mechanical planarization (CMP). This process enables full control of the polishing depth, sub-nanometer surface roughness, and wafer-scale uniformity of Si_3_N_4_ waveguide height with variation below 3%, critical for monolithic or heterogeneous integration of piezoelectric materials^64,65^, electro-optic materials^66^, and monolayer two-dimensional materials^67^. Afterward, the substrate is thermally annealed at 1200^∘^C to drive out the residual hydrogen impurities in the Si_3_N_4_ film^26,68^. A top SiO_2_ cladding composed of TEOS and low-temperature oxide is deposited on the wafer, followed by thermal annealing of the SiO_2_ at 1200 ^∘^C. Finally, the wafer is separated into chips via deep dry etching followed by dicing or backside grinding, to attain chip facets with the superior quality required for edge coupling^11,12^.

Figure 2b shows the final Si_3_N_4_ chips which contain multiple ring resonators of different free spectral ranges (FSRs). Figure 2c shows the optical micrograph of the Si_3_N_4_ microring resonator, bus waveguide, and filler patterns, as well as the tightly confined waveguide mode. The resulting negligible bending loss allows high-*Q* microresonators of small radii (below 23 *μ*m, i.e. 1 THz FSR), which find widespread applications in optical filters and coupled-resonator-based delay lines^69,70^. The filler patterns consist of horizontal and vertical bars uniformly distributed over the entire wafer area, and can significantly relax the as-deposited LPCVD Si_3_N_4_ film stress for crack prevention (see Supplementary Information). These filler patterns are also required for etching and CMP uniformity. In terms of the number of usable chips free from cracks, our fabrication yield is 100%, as no cracks have been observed in the past runs of more than 30 wafers using the same stress-release filler patterns.

### Statistical analysis of microresonator quality factors

**Fig. 3:**
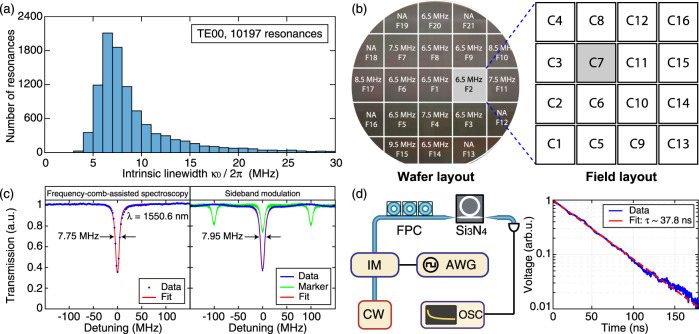
Statistical study of microresonator *Q* factors using multiple techniques. **a** Histogram of 10,197 TE_00_ resonances from twenty-six microresonators, showing the most probable value of *κ*_0_/2*π* = 6.5 MHz and *Q*_0_ = 30 × 10^6^. **b** DUV stepper exposure layout, and the most probable value *κ*_0_/2*π* of the C7 chips at different positions on the wafer. The reticle design containing sixteen chips is uniformly exposed in discrete fields over a 4-inch wafer. NA: not applicable, due to visible photoresist coating defects or missing C7 chips near the wafer edge. **c** Linewidth measurement of the same resonance at 1550.6 nm using frequency-comb-assisted diode laser spectroscopy (left, *κ*/2*π* = 7.75 MHz and *κ*_0_/2*π* = 5.87 MHz) and sideband modulation technique (right, *κ*/2*π* = 7.95 MHz and *κ*_0_/2*π* = 6.05 MHz). This resonance does not present a visible mode split. **d** Cavity ring-down measurement. An intensity modulator (IM) is used to switch off the pump field. The cavity ring-down signal is averaged 1000 times. The exponential fit gives an optical field decay time of *τ* = 37.8 ns, corresponding to a photon decay time of 18.9 ns and a loaded linewidth of *κ*/2*π* = 8.4 MHz. arb.u: arbitrary unit, AWG: arbitrary function generator, OSC: oscilloscope, CW: continuous-wave laser, FPC: fiber polarization controller.

We fabricate Si_3_N_4_ microresonators of 40 GHz FSR, 2200 nm width and 950 nm height, and systematically study the microresonator *Q* factors (i.e. losses). Frequency-comb-assisted diode laser spectroscopy^26,71^ is used to characterize the resonance frequency *ω*_opt_/2*π* and linewidth *κ*/2*π*, which relate to the resonance *Q* factor as *Q* = *ω*_opt_/*κ*. Here we mainly study the fundamental transverse electric (TE_00_) mode. The total (loaded) linewidth *κ*/2*π* = (*κ*_0_ + *κ*_ex_)/2*π*, the intrinsic loss *κ*_0_/2*π* and the coupling strength *κ*_ex_/2*π* are extracted from each resonance fit. Figure 3a shows the *κ*_0_/2*π* histogram of 10,197 TE_00_ resonances measured from twenty-six microresonators. The most probable value is *κ*_0_/2*π* = 6.5 MHz, corresponding to an intrinsic *Q* factor of *Q*_0_ = 30 × 10^6^. In comparison, *κ*_0_/2*π* = 9.5 MHz is found for the fundamental transverse magnetic (TM_00_) mode, corresponding to *Q*_0_ = 20 × 10^6^ (see Supplementary Information). In addition, by plotting the fitted *κ*_0_/2*π* as a function of the resonance wavelength measured from 1260 nm to 1630 nm, a weak trend showing a larger *κ*_0_/2*π* at a shorter wavelength is observed (see Supplementary Information). Finally, as the threshold power for soliton formation scales as 1/*Q*^2^, such a high microresonator *Q* allows soliton formation with 40 GHz repetition rate with only 10 mW optical power, obviating the need for an optical power amplifier (see Supplementary Information).

Next, we demonstrate the wafer-scale yield of our fabrication technology. Figure 3b shows our mask layout comprising 4 × 4 chip designs on the DUV stepper reticle. Each chip has a 5 × 5 mm^2^ size, and contains multiple microresonators as shown in Fig. 2b. The DUV stepper writes the reticle pattern uniformly over the full 4-inch wafer in discrete fields. The calibration chips of 40 GHz FSR studied here are the C7 chips. The most probable values of *κ*_0_/2*π* for the C7 chips are measured and plotted in each exposure field, as shown in Fig. 3b (see each individual histogram in Supplementary Information). In most fields, *κ*_0_/2*π* ⩽ 7.5 MHz is found. While exceptionally narrow linewidths have been reported previously in individual resonances, our statistics based on tens of thousands of analyzed resonances from dozens of samples at different wafer positions shows wafer-scale fabrication throughput and yield. In terms of loss values or microresonator *Q* factors, we define the yield as the ratio between the number of the chips with linewidths below 20 MHz (i.e. *Q*_0_ > 10 × 10^6^) to the total number of measured chips. In the present case of 40-GHz-FSR chips, the yield is calculated as 15/17 = 89% (The C7 chips in F12 and F13 are not available because of visible lithography defects; the C7 chips in F16, F18, F19, F21 are partially exposed, thus these four are not counted). In the Supplementary Information, the wafer-scale loss measurement is also illustrated on another wafer containing 10-GHz-FSR chips (used in ref. ^72^). Besides, a statistical process analysis based on fifteen wafers is shown, demonstrating the wafer-to-wafer reproducibility of our fabrication process.

In addition, a sideband modulation technique^73^ is used to measure the resonance linewidth *κ*/2*π* and to fit *κ*_0_/2*π*. Two sidebands, each separated from the carrier by 100 MHz, are used to calibrate the resonance linewidth. Figure 3c compares the measured *κ*/2*π* and fitted *κ*_0_/2*π* of the same resonance that does not present a visible mode split, using both the frequency-comb-assisted diode laser spectroscopy (*κ*/2*π* = 7.75 MHz and *κ*_0_/2*π* = 5.87 MHz) and the sideband modulation technique (*κ*/2*π* = 7.95 MHz and *κ*_0_/2*π* = 6.05 MHz). Both methods agree with each other, and show *Q*_0_ > 32 × 10^6^.

Furthermore, a cavity ring-down measurement is performed to validate the measured linewidth (see Methods). Figure 3d shows the schematic of the experimental setup and a representative ring-down measurement data. The fitted optical field decay time is 37.8 ns, corresponding to 18.9 ns photon storage time. The calculated loaded linewidth is *κ*/2*π* = 8.4 MHz, showing consistency between the three characterization methods used here.

### Meter-long spiral waveguides

In addition to high-*Q* microresonators, we also fabricate and characterize meter-long spiral waveguides that are key elements to build photonic true-time delay lines. Previously, silica suspended wedge waveguides^74^ and thin-core Si_3_N_4_ waveguides^51^ have been studied to build delay lines with losses below 0.1 dB m^−1^. However, in order to avoid bending losses, these waveguides must occupy more than 20 cm^2^ areas, thus requiring significant device footprints. While tight optical confinement can reduce the footprint, losses approaching even 1 dB m^−1^ have not been achieved in any nonlinear waveguide including thick-core Si_3_N_4_. Here, we demonstrate meter-long Si_3_N_4_ waveguides featuring ultralow loss and small footprint, which can enable key applications such as traveling-wave parametric amplifiers^56–59^, rare-earth-doped mode-locked lasers^60^ and optical coherence tomography (OCT)^75^.

**Fig. 4:**
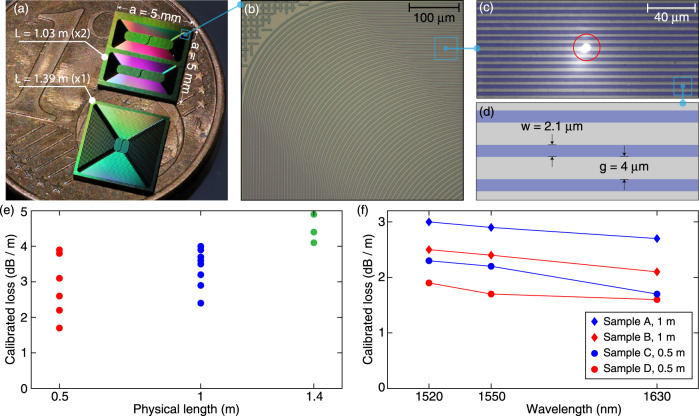
Small-footprint, meter-long, ultralow-loss Si_3_N_4_ spiral waveguides. **a** Photograph showing Si_3_N_4_ chips containing two 1.0-meter-long and one 1.4-meter-long spiral waveguides. **b**, **c** Optical micrographs of the densely packed Si_3_N_4_ waveguides in Archimedean spirals, taken with a yellow light camera (**b**) and IR camera (**c**). When 1550 nm laser light is coupled into the waveguide, light-scattering defects are observed under the IR camera (highlighted with a red circle). **d** Schematic showing the waveguide width and spacing. **e** Measured and calibrated optical losses at 1550 nm wavelength in 0.5 m, 1.0 m, and 1.4 m long spiral waveguides. **f** Measured and calibrated losses at different wavelengths for four selected samples.

Figure 4a shows a photograph of photonic chips containing Si_3_N_4_ waveguides of physical lengths *L* > 1 m. Figure 4b–d shows the spiral layout. The waveguides are densely packed in Archimedean spirals, with waveguide width *w* = 2.1*μ*m and gap distance *g* = 4*μ*m. Three lengths are studied here: a 0.5-meter-long spiral containing 50 coils and covering a 3.1 mm^2^ area; a 1.0-meter-long spiral containing 106 coils and covering a 6.6 mm^2^ area; and a 1.4-meter-long spiral containing 130 coils and covering a 20.2 mm^2^ area. Compared with the previous work based on thin-core Si_3_N_4_ waveguides^51^ showing a device footprint of more than 20 cm^2^ area for 1 meter physical length, our devices reduce the necessary footprint by a factor of 300, critical for photonic integration. Figure 4e shows the measured losses in multiple samples, calibrated using the adjacent 5-millimeter-long waveguide which has a fiber-chip-fiber through coupling efficiency of 33% (4.8 dB for two chip facets). The lowest-loss values found are 1.7 dB m^−1^ for 0.5 m length, 2.4 dB m^−1^ for 1.0 m length, and 4.1 dB m^−1^ for 1.4 m length. These loss values are higher than the value extrapolated from microresonator *Q* characterization (1.0 dB m^−1^). Meanwhile, the overall trend shows higher losses in longer waveguides. We attribute both observations to additional light-scattering defects. Light-scattering defects are found under an infrared (IR) microscope, as shown in Fig. 4c. By counting the number of defects in high-loss spirals, we estimate that each defect causes 1–2 dB extra loss. The defect probability depends on the waveguide area. These defects are likely caused by particle contamination on the wafer, as we have verified that these defects are not on the DUV reticle which would generate the same defects in the same position in each exposure field. Figure 4f shows the calibrated losses measured at different wavelengths for four selected samples. Again, a trend showing higher losses at shorter wavelengths is observed.

### Quantitative analysis of absorption losses using a Kerr-calibrated linear response measurement

Next, we quantitatively investigate the intrinsic absorption and scattering losses of our Si_3_N_4_ waveguides. The optical losses in the telecommunication band have two main contributions: Rayleigh scattering losses that are mainly caused by waveguide sidewall roughness, and absorption losses due to e.g. hydrogen impurities. While the hydrogen absorption loss can be efficiently eliminated via repeated thermal annealing of Si_3_N_4_ at high temperature (~1200 ^∘^C)^26,68^, efforts on loss reduction have mainly been focused on reducing waveguide roughness via optimized dry etching^25^ and etchless processes^76,77^. In addition, the large mode area of thin-core Si_3_N_4_ waveguides^49–51^ results in reduced optical mode interaction with waveguide sidewall roughness, and thereby reduced scattering losses.

**Fig. 5:**
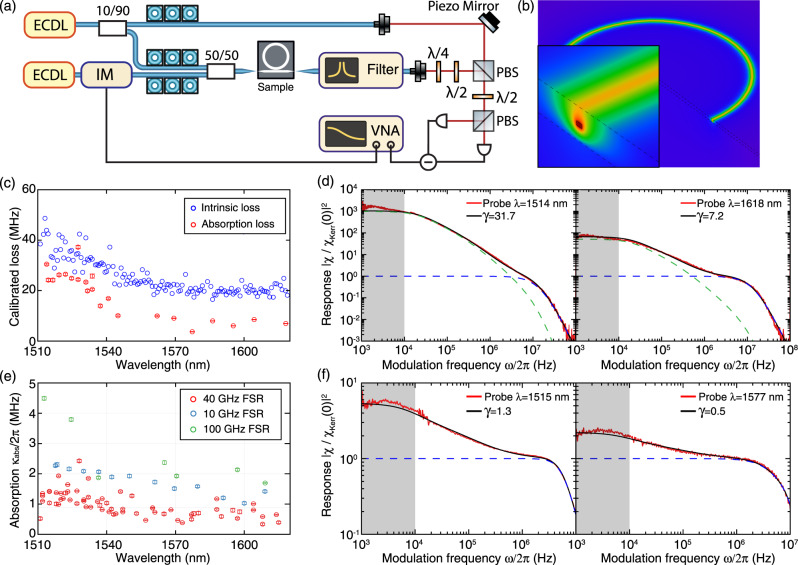
Probing the absorption loss of Si_3_N_4_ microresonators via Kerr-nonlinearity-calibrated thermal response measurements. **a** Experimental setup. ECDL: external-cavity diode lasers. IM: intensity modulator. VNA: vector network analyzer. PBS: polarization beam splitter. **b** Thermal simulation of the temperature distribution in the waveguide structures. **c** Comparison of the loss values measured using the response measurement and frequency-comb-assisted diode laser spectroscopy, on a partially annealed sample with prominent hydrogen absorption losses. This comparison reveals an approximate, wavelength-independent, 12 MHz loss difference between the two datasets, which is presumed to be due to intrinsic scattering loss. **d** For the two resonances at 1514 nm and 1618 nm shown in (**c**), the measured (red) and fitted (black) frequency response *χ*(*ω*) normalized to *χ*_Kerr_. The fitted cavity cutoff frequencies are, *κ*/4*π* = 22.7 MHz (*λ* = 1514 nm), and *κ*/4*π* = 17.1 MHz (*λ* = 1618 nm). **e** Calibrated absorption loss *κ*_abs_/2*π* of different resonances from different samples. **f** For the two resonances at 1515 nm and 1577 nm of the 40-GHz-FSR samples shown in (**e**), the measured frequency response *χ*(*ω*) normalized to *χ*_Kerr_ (red and black). The fitted cavity cutoff frequencies are, *κ*/4*π* = 7.4 MHz (*λ* = 1515 nm), and *κ*/4*π* = 10.8 MHz (*λ* = 1577 nm). In (**d**, **f**), we show the fitted Kerr (blue) and thermal (green) responses, and the gray areas mark the modulation frequency range from 1 to 10 kHz (the measured response within this range is not included in the response fitting). The fitted values of $\gamma =\frac{{\chi }_{\text{therm}}(0)}{{\chi }_{\text{Kerr}}(0)}$ are shown in (**d**, **f**). The fitted thermal response $| \frac{{\chi }_{{\rm{therm}}}(\omega )}{{\chi }_{{\rm{Kerr}}}(0)}{| }^{2}$ is not shown in (**f**) as it is mostly below 10^−1^. The error bars shown in (**c**, **e**) account for fitting errors (95% confidence interval).

To quantify the thermal absorption loss of our Si_3_N_4_ waveguides, a linear response measurement^36^ is performed. We characterize the resonance frequency shift response of the probe mode induced by intensity modulation of the pump mode, and utilize the measured response to calibrate the absorption loss of the pump resonance. The method exploits the fact that the thermal response *χ*_therm_(*ω*) dominates at low frequency, while the Kerr response *χ*_Kerr_(*ω*) dominates at higher frequency. The experimental setup is shown in Fig. 5a. The pump laser is first tuned to the optical resonance (frequency *ν*_*m*_) for which the thermal absorption loss *κ*_abs_/2*π* is to be characterized, and is then intensity-modulated at a frequency of *ω*/2*π* which is swept from 1 kHz to 100 MHz. Meanwhile, the probe laser is loosely locked (i.e. with ~300 Hz locking bandwidth) to a probe resonance whose frequency is $\nu ^{\prime}$.

This intensity modulation causes a change in the intracavity photon number *n*_c_ of the pump mode, which modulates the resonance frequency of the probe mode via Kerr and thermal nonlinearities. The pump power is maintained sufficiently low ${\mathcal{O}}(100\mu {\rm{W}})$, such that the steady-state frequency shift of the probe mode is small compared to the resonance linewidth *κ*/2*π*, i.e. $\delta {\nu }_{m^{\prime} }\ll \kappa /2\pi$. In this linear regime, the probe mode’s frequency response to the modulated pump power is given by^36^

1 $$\chi (\omega )=\frac{\delta {\nu }_{m^{\prime} }}{\delta {n}_{{\rm{ph}}}}={\chi }_{{\rm{therm}}}(\omega )+{\chi }_{{\rm{Kerr}}}(\omega ).$$

The total response *χ*(*ω*) consists of two parts: the Kerr response *χ*_Kerr_(*ω*) with infinite bandwidth, and the thermal response *χ*_therm_(*ω*) with a corner frequency around 40 kHz. Therefore, by calibrating the response *χ*(*ω*) as a function of the modulation frequency *ω*/2*π*, *χ*_therm_(*ω*) and *χ*_Kerr_(*ω*) can be individually identified. Using the inferred values of *χ*_therm_(*ω*) and *χ*_Kerr_(*ω*) at DC (*ω* = 0), the absorption rate is calculated as

2 $${\kappa }_{{\rm{abs}}}=\frac{2c{n}_{\text{mat}}{n}_{2}}{{n}_{g}{n}_{{\rm{eff}}}{V}_{{\rm{eff}}}\frac{{\rm{d}}T}{{\rm{d}}{P}_{{\rm{abs}}}}\frac{{\rm{d}}{n}_{\text{mat}}}{{\rm{d}}T}}\frac{{\chi }_{{\rm{therm}}}(0)}{{\chi }_{{\rm{Kerr}}}(0)}$$

where *V*_eff_ is the effective optical mode volume, *n*_2_ = 2.4 × 10^−19^m^2^/W is the nonlinear index of Si_3_N_4_, *n*_*g*_ = 2.1 is the group index, *n*_mat_ = 2.0 is the material index, *n*_eff_ = 1.8 is the effective index, d*n*_mat_/d*T* = 2.5 × 10^−5^/K is the thermo-optic coefficient of Si_3_N_4_, and *P*_abs_ is the absorbed power. The full derivation of Eq. (2) is shown in the Supplementary Information.

The frequency response $\delta {\nu }_{m^{\prime} }$ to the pump modulation is transduced into phase modulation of the probe laser. This phase modulation is measured using balanced homodyne detection, with the pump laser filtered out before detection (see “Methods”). To evaluate the absorption rate *κ*_abs_, the factor *γ* = *χ*_therm_(0)/*χ*_Kerr_(0) is retrieved by fitting the response function shown in Eq. (3) in “Methods”, which consists of one simulated thermal cutoff and two cavity cutoffs, to the measured response at frequencies above 10 kHz. This 10 kHz frequency cutoff for fitting is chosen to be far outside the locking bandwidth, and the thermal response simulation within this frequency range is still validated. Therefore, we actually anchor the response DC offset in Eq. (2) at 10 kHz. Finite-element simulations of optical mode profiles and bulk absorption heating (see Fig. 5b) are performed to calculate the coefficients *V*_eff_ and to retrieve the frequency-domain thermal response function $\frac{{\rm{d}}T}{{\rm{d}}{P}_{{\rm{abs}}}}(\omega )$ used in the fitting (see “Methods”). The simulation model we use to extract the thermal response function is validated by our recent measurement^78^ of the thermorefractive noise in these Si_3_N_4_ devices. Figure 5d, f presents four examples of measured and fitted *χ*(*ω*). Supplementary Information provides more details on data analysis.

To validate our method, we first benchmark the linear response measurement by characterizing a partially annealed Si_3_N_4_ sample whose resonance linewidth data have been published in ref. ^26^. We characterize this particular sample again, using both the response measurement and frequency-comb-assisted diode laser spectroscopy, and compare the results using both methods in Fig. 5c. Assuming a wavelength-independent scattering loss of 12 MHz, the measured absorption loss using the response measurement agrees with the total loss measured spectroscopically.

Figure 5e shows the calculated absorption rates *κ*_abs_/2*π* of different resonances from four 40-GHz-FSR Si_3_N_4_ samples featuring *Q*_0_ > 30 × 10^6^ (this work), in comparison with 10-GHz-FSR samples (used in ref. ^72^, *κ*_0_/2*π* = 8.5 MHz) and 100-GHz-FSR samples (used in ref. ^26^, *κ*_0_/2*π* = 13.5 MHz) fabricated using the same process but from different wafers. All samples show similar trends, and present two conclusions. First, the mean absorption loss for 40-GHz-FSR samples is *κ*_abs_/2*π* ~ 1 MHz, corresponding to an absorption-loss-limited *Q* factor of approximately 2 × 10^8^. Therefore, the optical losses of our Si_3_N_4_ waveguides (*κ*/2*π* = 6.5 MHz) are currently dominated by scattering losses. Second, for all the samples studied, *κ*_abs_/2*π* is higher around 1520 nm, compared to the value at e.g. 1600 nm. This is caused by the residual hydrogen impurities in our thermally annealed Si_3_N_4_. Note that only standard LPCVD Si_3_N_4_ /SiO_2_ films and thermal annealing are used in our fabrication to achieve such low absorption losses.

## Discussion

In conclusion, we have demonstrated a fabrication technology enabling high-yield and reproducible wafer-scale manufacturing of ultralow-loss, high-confinement, anomalous-GVD Si_3_N_4_ PIC. This optimized fabrication process employs standard CMOS foundry techniques, and has advantages in multiple aspects compared to previously reported Si_3_N_4_ fabrication processes (see the comparison chart in the Supplementary Information). We present a statistical study of microresonator losses based on tens of thousands of analyzed resonances. We introduce a novel method to determine the absorption losses, and use it to reveal that our waveguide losses are dominated by scattering losses, which could be further reduced via e.g. optimized lithography and etching. In the ideal case limited only by thermal absorption losses, the potential microresonator *Q* is calculated to exceed 2 × 10^8^ (corresponding to a linear loss of 0.15 dB m^−1^). The optimized photonic Damascene fabrication technology allows tight confinement, ultralow-loss, high-yield, meter-scale, nonlinear PIC. Transferring the present Si_3_N_4_ photonics technology to standard commercial foundries, and merging it with silicon photonics using heterogeneous integration technology^3–5^, will significantly expand the scope of today’s integrated photonics and seed new applications.

## Methods

### Cavity ring-down

An intensity modulator (IM) is used to rapidly switch off the pump field. The ring-down signal of the transmitted light is recorded by a 1-GHz-bandwidth low-noise photodetector. A 50-kHz square wave electrical drive signal is generated using a fast arbitrary waveform generator, ensuring that the light is switched off significantly faster than the resonance linewidth. The upper and lower voltage levels of the square wave are adjusted to match the maximum and minimum transmission voltage of the IM, such that the electrical overshoot and undershoot of the square wave signal do not alter the ring-down slope. Due to the finite extinction ratio of the IM, the residual pump field beats with the leakage of the intracavity field, producing a field ring-down signal which is affected by the detuning of the laser from the cavity mode resonances^79^. At small detunings (*κ* ≫ Δ), the effective ring-down rate is increased by the laser’s detuning from cavity resonance, and thus the directly inferred quality factor is less accurate than the sideband fitting result. Therefore, the ring-down results can only serve as a lower bound of the loaded *Q* factor of the measured resonances. The estimated loaded linewidth *κ*/2*π* = 8.4 MHz is in agreement with the sideband fitting results, showing consistency between the three characterization methods used here.

### Thermal simulations

We use COMSOL Multiphysics to simulate the thermal response due to bulk absorption heating of our Si_3_N_4_ waveguides. The main material property coefficients of interest used in the current simulation are identical to the ones used in ref. ^78^ for simulating the Si_3_N_4_ thermorefractive noise. The thermo-optic coefficient^80^ of Si_3_N_4_, d*n*_mat_/d*T* = 2.5 × 10^−5^ K^−1^, is used here. We first simulate the waveguide optical mode profile (TE_00_ mode), from which the effective mode volume *V*_eff_ is calculated. Bulk absorption heating is introduced whose power distribution is proportional to the intensity distribution of the optical mode *ν*_*m*_. By solving the frequency-domain heat transfer equation, the response of the effective temperature to the modulated absorbed power, $\frac{{\rm{d}}T}{{\rm{d}}{P}_{{\rm{abs}}}}(\omega )$, is retrieved from a Fourier frequency sweep. The combined value of *V*_eff_ ⋅ d*T*/d*P*_abs_ is calculated as 3.60 × 10^−13^ K m^3^ W^−1^ in the case of full SiO_2_ cladding for samples used in Fig. 5e, f, and is 4.63 × 10^−13^ K m^3^ W^−1^ in the case without top SiO_2_ cladding for samples used in Fig. 5c, d.

### Response calibration

In order to extract the actual microresonator response *χ*(*ω*) from the experimentally photodetected $\chi ^{\prime} (\omega )$, the frequency response ${\chi }_{\det }(\omega )$ of our entire experiment setup and detection chain needs to be calibrated first. This is realized by direct detection of the pump power modulation $\delta P(\omega )\propto {\chi }_{\det }(\omega )$ in the absence of the probe laser and the pump filter. The measured response $\chi ^{\prime} (\omega )$ is normalized to the setup response ${\chi }_{\det }(\omega )$, and thus the actual microresonator response $\chi (\omega )=\chi ^{\prime} (\omega )/{\chi }_{\det }(\omega )$ is retrieved, with a constant factor. This constant factor is removed when retrieving *χ*_therm_(0)/*χ*_Kerr_(0) from the fitting of *χ*(*ω*) using a fitting function

3 $$\chi (\omega )={\chi }_{{\rm{Kerr}}}(0)\cdot (1+\frac{{\chi }_{{\rm{therm}}}(0)}{{\chi }_{{\rm{Kerr}}}(0)}\frac{{\chi }_{{\rm{therm}}}(\omega )}{{\chi }_{{\rm{therm}}}(0)})\frac{1}{1+2{\rm{i}}\omega /{\kappa }_{{\rm{probe}}}}\frac{1}{1+2{\rm{i}}\omega /{\kappa }_{{\rm{pump}}}}$$

with *κ*_probe, pump_/4*π* being the cavity cutoff frequencies for the pump and probe fields, respectively. The normalized thermal response function *χ*_therm_(*ω*)/*χ*_therm_(0) used in the fitting is retrieved from COMSOL simulation. In Fig. 5d, f, only the normalized response *χ*(*ω*)/*χ*_Kerr_(0) is shown, with the constant factor removed.

## Supplementary information

Supplementary Information

## Data Availability

The data that support the plots within this manuscript and other findings of this study are available on Zenodo (10.5281/zenodo.4273990). All other data used in this study are available from the corresponding authors upon reasonable request.
