# Supplementary material for: High-yield, wafer-scale fabrication of ultralow-loss, dispersion-engineered silicon nitride photonic circuits
Source: Nat Commun. 2021 Apr 16;12:2236. doi: 10.1038/s41467-021-21973-z (PMC8052462; doi:10.1038/s41467-021-21973-z)
Supplement: Supplementary file 1 — Supplementary Information [file 41467_2021_21973_MOESM1_ESM.pdf]

Supplementary Information for: High-yield, wafer-scale fabrication of ultralow-loss, dispersion-engineered silicon nitride photonic circuits

Junqiu Liu et al.

Supplementary Note 1. Statistical analysis of the TM_{00} resonance linewidths

Supplementary Figure 1 shows the intrinsic loss $\kappa_0/2\pi$ histogram of 4026 resonances, for the fundamental transverse magnetic (TM_{00}) mode. The most probable value of $\kappa_0/2\pi$ is 9.5 MHz, corresponding to $Q_0 = 20 \times 10^6$, lower than the $Q_0 = 30 \times 10^6$ of the TE_{00} mode.

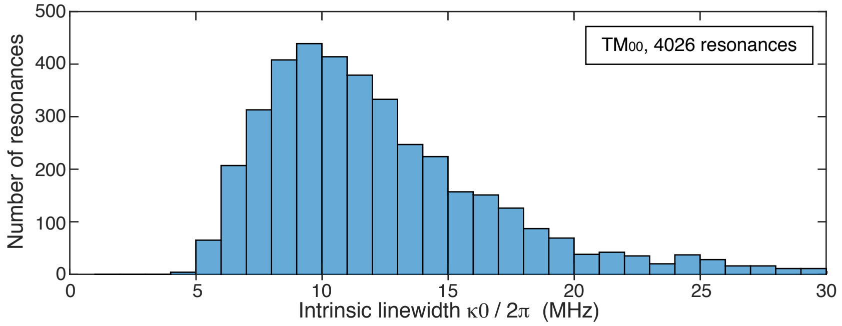

Supplementary Figure 1: The $\kappa_0/2\pi$ histogram for the TM_{00} mode. The most probable value is 9.5 MHz, corresponding to $Q_0 = 20 \times 10^6$.

Supplementary Note 2. Linewidth histograms in each individual field

Supplementary Figure 2 shows the $\kappa_0/2\pi$ histograms of the C7 chip (40 GHz FSR) in each stepper exposure field on the 4-inch wafer. The most probable values of $\kappa_0/2\pi$ are shown in Fig. 2(b) in the main manuscript.

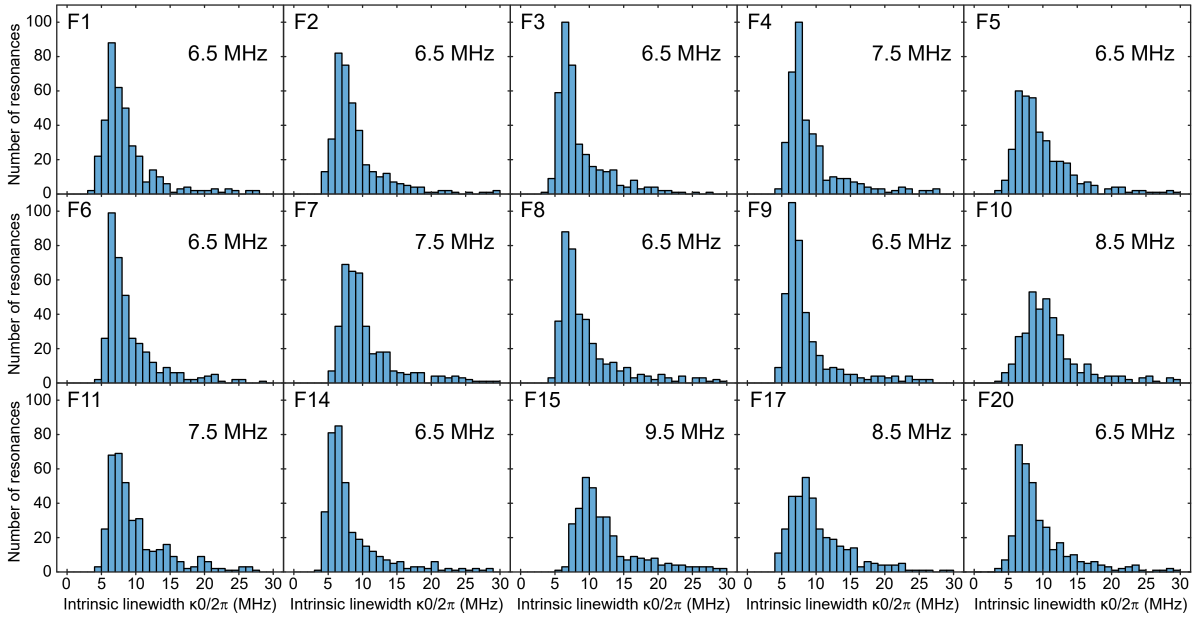

Supplementary Figure 2: The $\kappa_0/2\pi$ histograms of multiple 40-GHz-FSR chips shown in Fig. 2(b) in the main manuscript.

Supplementary Note 3. Statistical process analysis of multiple wafers

Wafer-scale distribution of resonance linewidths on a 10-GHz-FSR wafer: In the main manuscript, Fig. 2(b) shows the wafer map of 40-GHz-FSR chips' Qs in each stepper exposure field. Here we show that a high Q is also obtained reproducibly over the full 4-inch wafer scale, with 10-GHz-FSR chips. Supplementary Figure 3(c) shows our mask layout constituting 4×4 chip designs on the DUV stepper reticle. Each chip contains only a single 10-GHz-FSR microresonator. Supplementary Figure 3(a) shows that the DUV stepper uniformly exposes the reticle pattern over the full 4-inch wafer scale in discrete fields. The calibration chips studied here are the C15 chips. The most probable values of $\kappa_0/2\pi$ histograms of these C15 chips are measured and plotted in each exposure field, as shown in Supplementary Fig. 3(b). In most fields, $\kappa_0/2\pi \leq 9.5$ MHz is found.

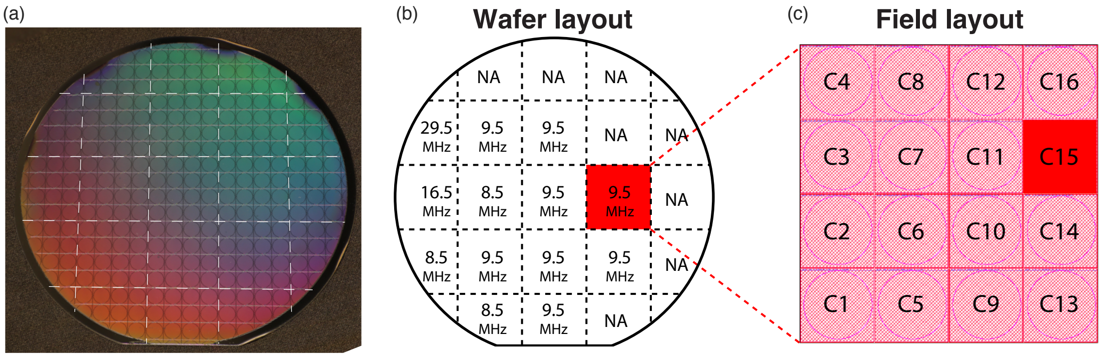

Supplementary Figure 3: Loss distribution on a 4-inch wafer consisting of 10-GHz-FSR chips. (a) Photo of the 4-inch wafer. (b) DUV stepper lithography exposure layout, and the most probable value $\kappa_0/2\pi$ of the C15 chips at different exposure fields. (c) The reticle design contains sixteen chips and is uniformly exposed in discrete fields on the wafer. NA: not applicable, due to visible photoresist coating defects or the design missing in particular fields close to the wafer edge.

Statistical analysis of process reproducibility: Using the same chip characterization and analysis methods, multiple wafers fabricated using the same process but at different times in our university cleanroom have been measured, as listed in Supplementary Table I. The intrinsic Q_0 is summarized from multiple chips' histograms. Quality factors of $Q_0 > 10 \times 10^6$ have been achieved in all fabricated wafers. Some wafers have been used in our published works. We note that Supplementary Table I only lists the wafers whose fabrication runs were smooth and had no error reported during the processing. Operation of our process in a foundry could significantly enhance the stability, reproducibility and even the performance of our wafer fabrication.

Supplementary Table I: Statistical analysis of process reproducibility based on multiple wafers fabricated at different times. $w \times h$: waveguide cross-section, width $\times$ height.

Batch	Release date	Intrinsic Q_0 ($\times 10^6$)	Cross-section $w \times h$ (μm^2)	FSR (GHz)	Note
# 1	May 2018	15	1.5×0.85	100	Used in Ref.[1,2].
	Aug. 2018	15	1.5×0.75	100	Used in Ref.[3,4].
# 2	Nov. 2018	30	2.1×0.82	100	
# 3	Mar. 2019	17	2.2×0.82	100	No reflow.
# 4	Apr. 2019	17	1.7×0.95	20	Used in Ref.[5].
# 5	Apr. 2019	22	2.1×0.95	10	Used in Ref.[5].
	May 2019	22	2.1×0.95	10	
# 6	June 2019	22	2.2×0.80	230	
# 7	July 2019	17	1.8×0.90	191	Used in Ref.[6].
# 8	Aug. 2019	30	2.2×0.95	40	Used in this work.
	Sept. 2019	30	2.2×0.95	40	Used in this work.
# 9	Feb. 2020	22	2.2×0.90	12	
# 10	May. 2020	17	2.2×0.90	50	
	June 2020	17	2.2×0.90	50	
# 11	Aug. 2020	10	2.1×0.65	100	No top SiO_2 cladding.

Supplementary Note 4. 40 GHz single soliton generation without EDFA

Using the Si_3N_4 microresonators featuring $Q_0 = 30 \times 10^6$ and anomalous group-velocity dispersion (GVD), here we demonstrate soliton microcomb generation at 40.6 GHz repetition rate, with only 10.2 mW optical power on chip (16 mW input power in the fiber), without using an erbium-doped fiber amplifier (EDFA).

The microresonator transmission trace from 1500 nm to 1630 nm is obtained using the frequency-comb-assisted diode laser spectroscopy[7] with one external-cavity diode lasers (ECDL, Santec TSL-510) that can scan the laser wavelength continuously (i.e. mode-hop-free)[8]. The precise frequency of each data point is calibrated using a commercial femtosecond optical frequency comb with 250 MHz repetition rate. For the TE_{00} mode family, the FSR of the microresonator and the anomalous GVD are extracted from the calibrated transmission trace by identifying the precise

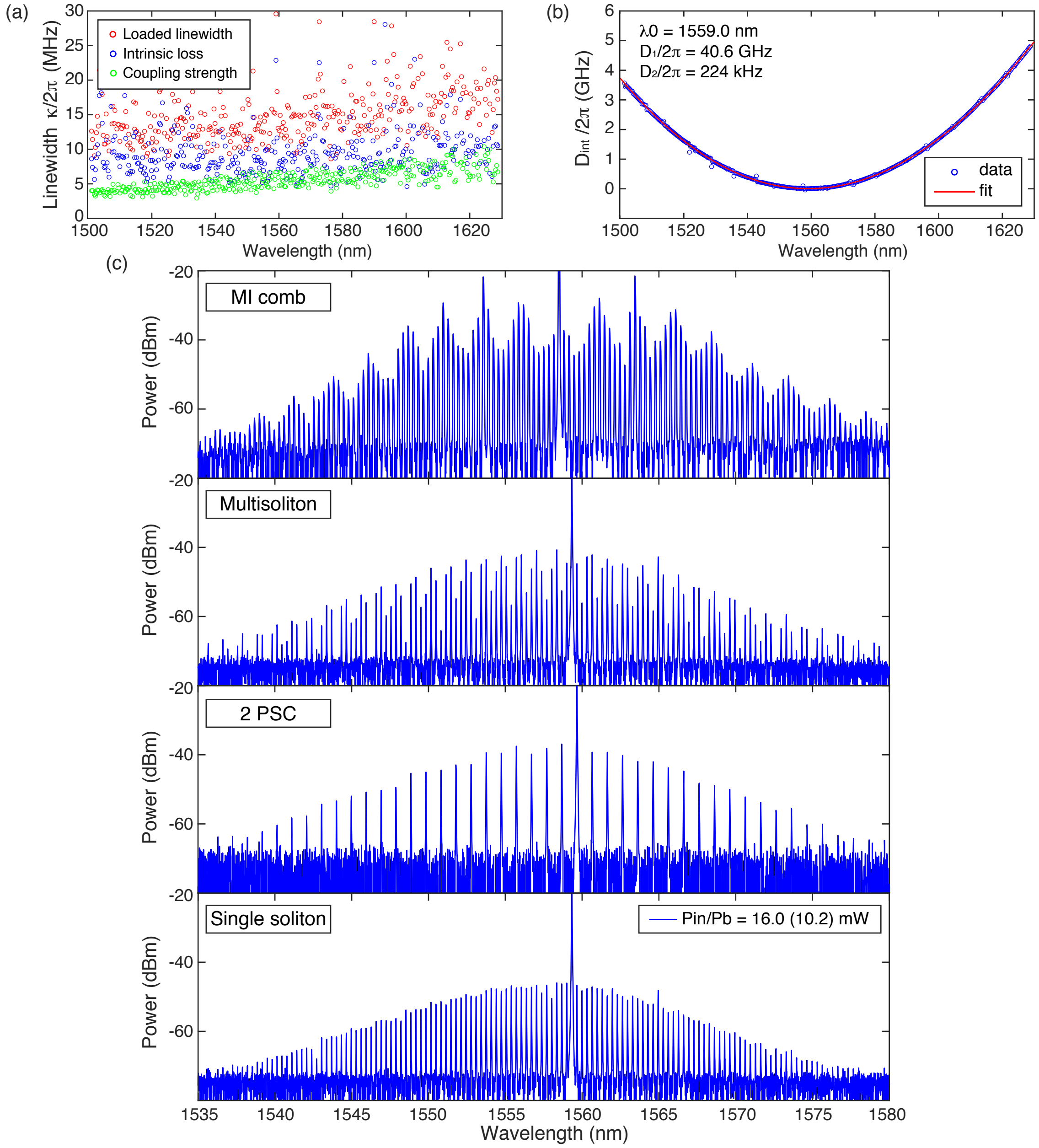

Supplementary Figure 4: Direct generation of a single soliton with 40.6 GHz repetition rate, using only a diode laser without optical power amplification. (a) Loaded linewidth, intrinsic loss, and coupling strength of each TE_{00} resonance from 1500 nm to 1630 nm. (b) Measured microresonator integrated dispersion. (c) Different comb states generated in the same device, including the MI comb, multi-soliton, two-soliton perfect soliton crystal (2 PSC), and the single soliton. The single soliton spectrum is generated with the input pump power of $P_{\text{in}} = 16.0$ mW ($P_{\text{b}} = 10.2$ mW on the chip).

frequency of each resonance. The total (loaded) linewidth $\kappa/2\pi = (\kappa_0 + \kappa_{\text{ex}})/2\pi$, the intrinsic linewidth (intrinsic loss) $\kappa_0/2\pi$ and the coupling strength $\kappa_{\text{ex}}/2\pi$ are extracted from each resonance fit[9,10].

Supplementary Figure 4(a) shows the measured linewidth of each TE_{00} resonance in a critically coupled microresonator. Supplementary Figure 4(b) shows the measured microresonator integrated dispersion $D_{\text{int}}/2\pi$. The FSR is $D_1/2\pi = 40.6$ GHz, and the GVD is $D_2/2\pi = 224$ kHz, obtained from fitting the measured GVD profile. Different comb states, including the modulation-instability (MI) comb, multi-soliton, perfect soliton crystal (PSC), and the single soliton, are generated in the same device. Using only a diode laser without an EDFA, the single soliton state is accessed with 10.2 mW power on the chip (input pump powers of $P_{\text{in}} = 16.0$ mW), as shown in Supplementary Fig. 4(c). The single soliton state is accessed via only laser piezo frequency tuning[11,12], and does not require complex soliton tuning methods.

Supplementary Note 5. Broadband linewidth measurement

The wavelength range of measured resonances can be extended using the frequency-comb-assisted *cascaded* diode laser spectroscopy[8] with three ECDLs covering different wavelength ranges (1260–1360 nm, 1355–1505 nm, and 1500–1630 nm). Supplementary Figure 5(a) shows the measured and fitted intrinsic loss $\kappa_0/2\pi$ from 1260 to 1630 nm. An overall trend showing a larger $\kappa_0/2\pi$ at a shorter wavelength is observed. Supplementary Figure 5(b) shows the $\kappa_0/2\pi$ histogram of 6851 TE_{00} resonances collected from five devices. The most probable value is shifted to $\kappa_0/2\pi = 9.5$ MHz, corresponding to $Q_0 = 20 \times 10^6$.

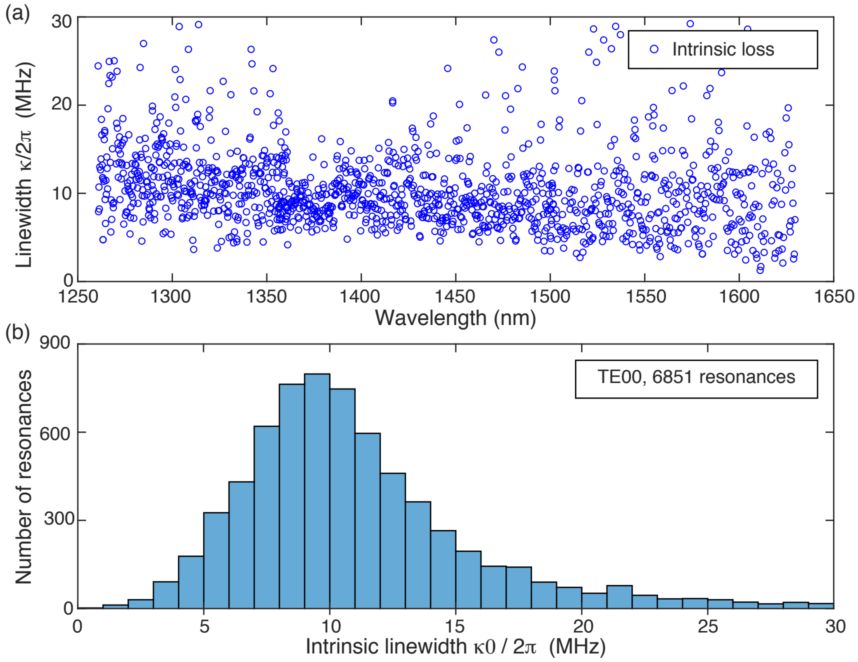

Supplementary Figure 5: Broadband linewidth measurement and analysis. (a) Measured resonance linewidth as a function of the wavelength from 1260 to 1630 nm. An overall trend showing a larger $\kappa_0/2\pi$ at a shorter wavelength is observed. (b) The $\kappa_0/2\pi$ histogram of 6851 TE_{00} resonances. The most probable value is $\kappa_0/2\pi = 9.5$ MHz, corresponding to $Q_0 = 20 \times 10^6$.

Supplementary Note 6. Reflow's impact on loss

In the photonic Damascene process, after dry etching, the patterned SiO_2 preform is thermal-annealed at 1250°C over its glass transition temperature. This allows to reflow the thermal wet SiO_2[13], in order to reduce the surface roughness introduced by the dry etching. The reflow step is performed in a standard silicon carbide atmospheric-pressure CVD tube. Two-times improvement in the microresonator Q factors has been reported in Ref.[13], which has been attributed to this preform reflow technique, however with a deformation of the waveguide cross-section as a trade-off. Here the impact of preform reflow on Q factors is studied in our high-Q microresonator fabricated with the

optimized Damascene process.

Supplementary Figure 6 compares the $\kappa_0/2\pi$ histograms of the TE_{00} mode, for 1500 nm waveguide width, with and without the preform reflow. The reflow was implemented at 1250 $°C$ for 24 h, same as reported in Ref.[13]. Without the reflow, the most probable $\kappa_0/2\pi = 15.5$ MHz is only marginally larger than the value with the reflow ($\kappa_0/2\pi = 14.5$ MHz). We attribute this to the improved lithography and dry etching in the current fabrication process, which have resulted in better waveguide sidewall quality and reduced roughness. Therefore the efficacy of reflow is reduced in high-Q microresonators, and might not be necessary.

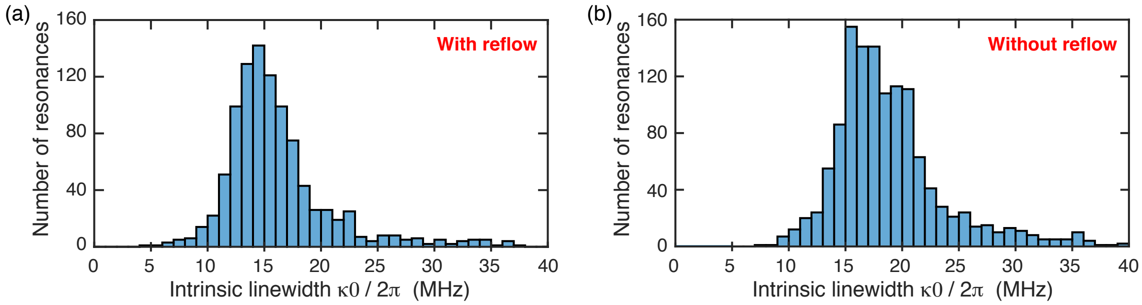

Supplementary Figure 6: Comparison of $\kappa_0/2\pi$ histograms in the cases with and without the preform reflow. (a) With 24 h reflow, the histogram of 1031 TE_{00} resonances shows the most probable value $\kappa_0/2\pi = 14.5$ MHz. (b) Without the reflow, the histogram of 1301 TE_{00} resonances shows the most probable value $\kappa_0/2\pi = 15.5$ MHz.

Despite the fact that the reflow can increase Q, it also deforms the waveguide cross-section, leading to a slanted sidewall from 90° to 98° angle as shown in Ref.[13]. This deformation causes difficulties in the control of critical dimensions. However, by reducing the reflow time to only 3 hours, the sidewall slant effect can be significantly reduced. In the main manuscript, Fig. 1(b) has shown nearly maintained sidewall angles with 3 h reflow instead of 24 h. All the 40- and 10-GHz-FSR high-Q chips shown in this work were fabricated with 3 h reflow time.

Supplementary Note 7. Etchback planarization

The etchback planarization process consists of dry etching and chemical-mechanical polishing (CMP). Supplementary Figure 7(a, b) shows the process flow and the SEM images of each step. After LPCVD Si_3N_4 deposition,

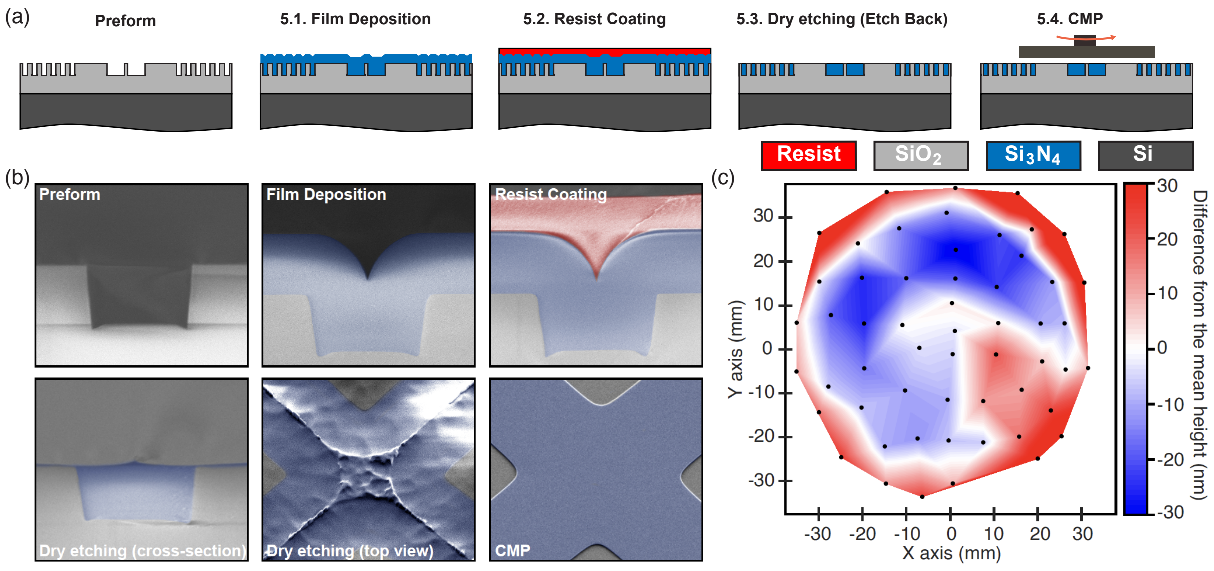

Supplementary Figure 7: Process flow and results of etchback planarization. (a) Etchback process flow. (b) SEM images of the wafer cross-section / tilted top view at different steps. (c) The final Si_3N_4 waveguide height measured at different positions on a 4-inch wafer. The height map is plotted in such a way that the height difference from the mean height is shown.

continuous Si_3N_4 films are coated on the wafer's frontside and backside. The wafer is then coated with common photoresist (PR) on the frontside. Depending on the PR viscosity, the spin-coating speed and the waveguide width, a proper PR thickness is needed for sufficient coating conformality. In our case, 600 nm PR is coated on the wafer, followed by PR reflow, to achieve a flat wafer top surface. Then a dry etching with an etch selectivity of Si_3N_4 : PR : SiO_2 =1 : 1 : 1 is performed, to uniformly remove the excess Si_3N_4 together with the PR. In the recipe, adding O_2 increases the PR etch rate without affecting the Si_3N_4 etch rate. Therefore, the etch rates of Si_3N_4 and PR can be controlled independently. Supplementary Figure 8(a) shows the etch rates of Si_3N_4 and PR as a function of the O_2 flow. End point detection (EPD) during dry etching signals clear transitions from PR to Si_3N_4, and from Si_3N_4 to SiO_2. Thus, the dry etching enables precise control of the etch depth and the amount of materials removed, compared to the common CMP process where EPD is usually absent or difficult to implement. As a result, the etchback can be critically stopped when all the excess Si_3N_4 is removed.

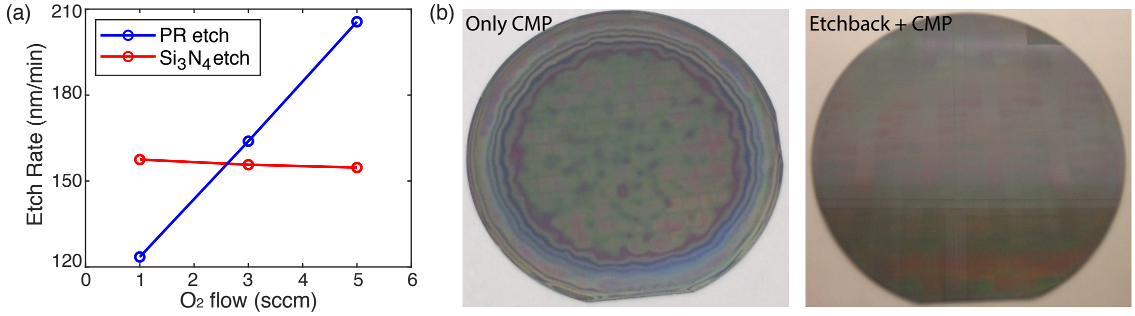

Supplementary Figure 8: Etchback etch rate control, and comparison of wafer images using different planarization processes. (a) When increasing the O_2 flow, the Si_3N_4 etch rate is nearly unchanged, while the photoresist (PR) etch rate increases linearly. When the O_2 flow is around 2.5 SCCM (Standard Cubic Centimeters per Minute), the etch rates for Si_3N_4 and PR are equal (≈157 nm/min). (b) Comparison of the wafer images using only CMP and CMP combined with etchback. The visible color patterns are due to natural light interference in thin films, caused by the SiO_2 thickness variation on the wafer.

Next, the wafer's backside Si_3N_4 is removed by dry etching, to reduce the wafer bow[14]. The measured bow value of the wafer frontside using a laser interferometer is below 5 μm, indicating that the wafer is sufficiently flat. The reason to perform the frontside etchback before the backside Si_3N_4 etch is to avoid potential crack formation during the wafer transfer and clamping in the dry etcher.

The etchback process creates a wafer top surface which is flat but not smooth. A short CMP step, removing only a thin layer of materials (less than 50 nm), is already sufficient to reduce the surface roughness to sub-nanometer levels (measured using an atomic force microscopy as shown in Ref.[13]). All the excess Si_3N_4 has been removed during the etchback and backside etch, resulting in a small wafer bow below 5 μm. Therefore, the CMP's polishing rate and uniformity can be easily calibrated. This final CMP step serves as a fine control of the waveguide height. Supplementary Figure 7(c) shows the measured Si_3N_4 waveguide height in different places on a full 4-inch wafer, using a reflectometer (Nanospec M6100). The measured waveguide height map shows ± 30 nm variation, corresponding to ±3% of 950 nm waveguide height, a value comparable to typical LPCVD Si_3N_4 deposition uniformity. We note that our current height uniformity is limited by both the CMP and the etchback (dry etching). The height variation in the radial direction (i.e. center is thinner, edge is thicker) is caused by the CMP / photoresist coating (as a result of the edge effect). The height variation showing that the right-bottom is thicker is caused by the etchback, as the wafer chuck of our dry etcher has a non-uniform temperature distribution which introduces an etch-rate variation over the 4-inch wafer scale. To further improve the height uniformity, it is preferred to use larger wafers (as the edge effect is effectively weaker), and a dry etcher with a wafer chuck of a uniform temperature distribution.

Supplementary Figure 8(b) compares the photographs of two wafers, one prepared with only the CMP and the other with combined etchback and CMP. The visible color patterns are due to natural light interference, caused by the SiO_2 thickness variations on the wafer. It is clear that the combination of etchback and CMP gives better thickness uniformity over the wafer scale. This process enables full control of polishing depth, sub-nanometer surface roughness (see Ref.[13]), and wafer-scale uniformity of Si_3N_4 waveguide height with 3% variation. Based on this process, monolithic or heterogeneous integration of piezoelectric aluminium nitride actuators (Ref.[6,15]), electro-optic lithium niobate modulators (Ref.[16]) and metallic heaters (Ref.[17]) has been demonstrated.

Furthermore, to verify the wafer-scale planarization uniformity, we measure the microresonator GVD parameter ($D_2/2\pi$) of each 40-GHz-FSR samples (C7), as shown in Supplementary Fig. 9. Note that the wafer-scale uniformity of the most probable value $\kappa_0/2\pi$ of the C7 chips has been already shown in Fig. 3(b) in the main manuscript. In Supplementary Fig. 9(a), the minimum and maximum values of $D_2/2\pi$ in the center 9 fields (F1 – F9) are 204 kHz and 221 kHz, respectively. Each field occupies 20.24×20.24 mm^2 area, thus the center 9 fields occupy 60.72×60.72

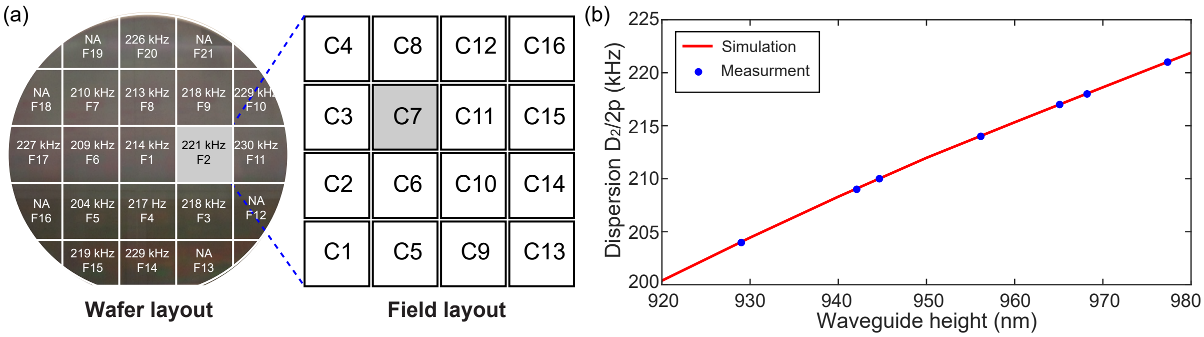

Supplementary Figure 9: Characterization of the wafer-scale uniformity of the microresonator GVD parameter $D_2/2\pi$. (a) Wafer-scale uniformity of $D_2/2\pi$. The minimum and maximum values of $D_2/2\pi$ in the center 9 fields (F1 – F9) are 204 kHz and 221 kHz, respectively. (b) COMSOL FEM simulation of $D_2/2\pi$ as a function of the waveguide height (red line). The $D_2/2\pi$ values of Fields F1 – F9 are also marked (blue dots). As seen, $D_2/2\pi$ variation from 204 kHz to 221 kHz corresponds to 930 nm to 980 nm waveguide height variation. This 50 nm height variation agrees with the measured planarization uniformity.

mm^2 area. From a finite-element method (FEM) simulation using COMSOL Multiphysics, $D_2/2\pi$ variation from 204 kHz to 221 kHz corresponds to 930 nm to 980 nm waveguide height variation. This 50 nm height variation agrees with Supplementary Fig. 7(c) that shows 60 nm height variation over a circular area of 60 mm diameter. On the wafer edge, due to the edge effect of LPCVD, etchback and CMP, $D_2/2\pi$ is larger as the waveguide height is larger.

Supplementary Note 8. Stress release with filler patterns

We have not observed any cracks in more than 30 wafers fabricated using the current process. The stress-release filler patterns extending to the wafer edge significantly prevent crack formation starting from the wafer edge. The design criteria of stress-release filler patterns are:

- The filler pattern should contain the same structure and density in the horizontal and vertical directions. As shown in Ref.[18], if only horizontal bars are used, cracks are likely to form in the vertical direction. The horizontal bars, which create film discontinuity of LPCVD Si_3N_4 in the vertical direction, relax the film stress in the vertical direction. Therefore, only cracks in the vertical direction are generated as a result of accumulated horizontal stress.

- The filler pattern should have sufficient density, such that the film stress does not accumulated over a larger area of continuous film. Ideally, the higher the density is, the better the stress release is. In our case, the choice of a moderate density of filler patterns is to account not only the stress release but also the dry etching and CMP uniformity.

- The filler pattern used in our current process consists of horizontal and vertical bars, forming "#" structures. The bar is a 2×20 μm^2 rectangle. The choice of 2 μm width is to match the typical width of the main *functional* waveguides (i.e. bus waveguides and microrings) which are between 1.5 μm to 2.5 μm; The choice of 20 μm length is to match the pattern density of the main functional waveguides with an exclusion zoom. It should also be mentioned that, the bar width should not be much smaller than $2h$, where h is the thickness of the deposited Si_3N_4 film (in our case, h is around 1000 nm). The reason is that, the LPCVD Si_3N_4 growth on the substrate is *conformal*[18,19], i.e. the film grows not only from the bottom of the etched trench but also from the sidewall. Therefore, if the bar width is much smaller than $2h$, the conformal deposition of LPCVD Si_3N_4 can completely fill the filler pattern trenches and form a continuous film, resulting in continuously accumulated stress that can cause cracks. The overall filling ratio of the current "#" filler patterns is approximately 24% in our design.

- There is no filler pattern applied in the coverage area of meter-long spiral waveguides. However, still no cracks are formed due to the fact that the filling ratio of the functional waveguides in this design is approximately 34%, sufficiently high for crack prevention. Therefore, the design of stress-release filler patterns is highly flexible: In the design of *sparse* functional waveguides, filler patterns can be placed in the available open area; In the design of *dense* functional waveguides that already provide sufficient spatial topography for stress release, no filler pattern is needed.

Supplementary Note 9. Waveguide layout designs

Supplementary Figure 10 shows the GDS design layouts of microring resonators of 10, 40, and 100 GHz FSRs, on 5×5 mm^2 chips. The microresonator is coupled to a bus waveguide whose waveguide width is identical to the microresonator's waveguide width, to achieve high coupling ideality[20]. For FSRs below 40 GHz, the rings are densely packed on the chip and the space is fully used. Thus the maximum number N of the microresonators on the wafer is approximately calculated as $N \approx A_0/A_r$, where A_0 is the wafer area (for the 4-inch wafer, $A_0 \approx 63$ cm^2, calculated with an effective radius of 4.5 cm) and A_r is the area of the microresonator for a given FSR.

For FSRs above 100 GHz, the space is currently not fully used on the 5×5 mm^2 chip. In principle, the design density can be significantly increased by making the chip smaller (e.g. 2×2 mm^2). The 5×5 mm^2 chip size chosen here is to facilitate the manual handling of chips with tweezers, not to increase the pattern density.

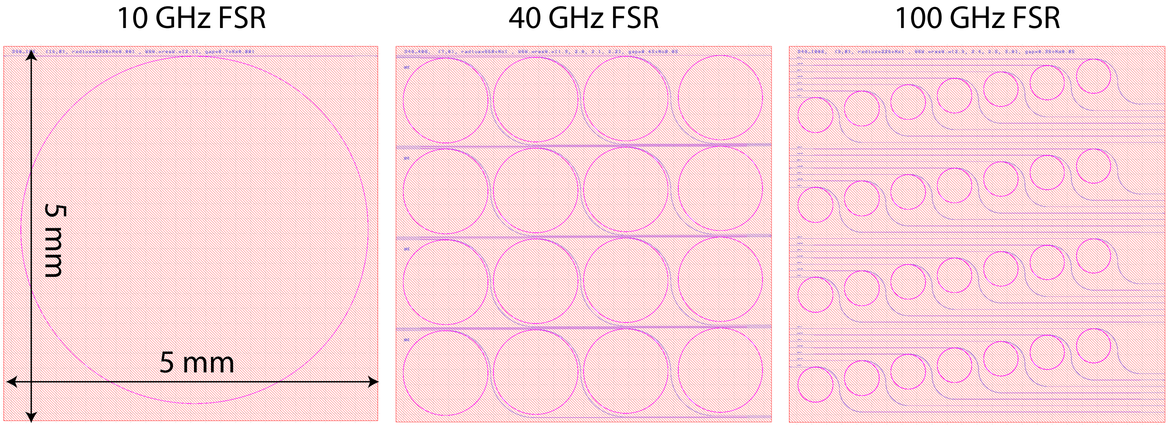

Supplementary Figure 10: GDS design layouts of microring resonators of 10, 40, and 100 GHz FSRs, on 5×5 mm^2 chips.

For meter-long spirals, the design density is shown in the Fig. 4 in the main manuscript. In the currently case, the separation distance between waveguides is 4 μm. The minimum distance depends on the mode coupling between adjacent waveguides. Based on our experiments and eigenmode simulations, the minimum distance can be further reduced to less than 2.5 μm.

Supplementary Note 10. Comparison of silicon nitride fabrication processes

Supplementary Table II compares several Si_3N_4 fabrication processes[5,24–30] developed and reported recently. These processes use mainly LPCVD Si_3N_4, except Ref.[27] uses PECVD Si_3N_4. Both photolithography and electron-beam lithography (EBL) have been used. The wafer sizes range from 3 inch to 8 inch.

Supplementary Table II: Comparison of existing Si_3N_4 fabrication processes. $w \times h$: waveguide cross-section, width $\times$ height. DUV: Deep-UV photolithography. UV: UV photolithography. EBL: electron-beam lithography.

Process	Wafer size	Lithography	Intrinsic Q_0 ($\times 10^6$)	Cross-section $w \times h$ μm^2	FSR (GHz)	GVD at 1550 nm
Damascene (this work)	4-inch	DUV	30 (with statistics)	2.2×0.95	40	Anomalous
Damascene[5]	4-inch	DUV	23 (with statistics)	2.1×0.95	9.8	Anomalous
Subtractive[24]	4-inch	EBL	17	3.0×0.60	24.7	Normal
Subtractive[25]	4-inch	EBL	37	2.5×0.73	200	Anomalous
Subtractive[26]	3-inch	EBL	11 (with statistics)	2.0×0.74	100	Anomalous
Subtractive[27]	3-inch	EBL	1.6 (with statistics)	2.0×0.92	130	Anomalous
Subtractive[28]	8-inch	DUV	5 (with statistics)	1.6×0.80	200	Anomalous
Subtractive[29]	4-inch	UV	2 (with statistics)	2.0×0.83	200	Anomalous
Subtractive[30]	4-inch	UV	81	11×0.04	3.3	Normal

Supplementary Note 11. Derivation of response relation

In the linear regime, the frequency response $d\nu_{m'}$ to the modulated pump power dn_c (in the microresonator) at modulation frequency $\omega/2\pi$ is given by

$$\chi(\omega) = \frac{d\nu_{m'}}{dn_\mathrm{c}} = \chi_\mathrm{therm}(\omega) + \chi_\mathrm{Kerr}(\omega) \tag{1}$$

In the DC modulation regime ($\omega \to 0$), the Kerr response term $\chi_\mathrm{Kerr}(0)$ is calculated[21] as

$$\begin{aligned}\chi_\mathrm{Kerr}(0) &= \frac{d\nu_{m',\mathrm{Kerr}}}{dn_\mathrm{c}} \\ &= \frac{d\nu_{m',\mathrm{Kerr}}}{\nu_{m'}} \cdot \frac{\nu_{m'}}{dn_\mathrm{c}} \\ &= \frac{2n_2}{n_\mathrm{eff}} dI \cdot \frac{\nu_{m'}}{dn_\mathrm{c}} \\ &= \frac{2n_2}{n_\mathrm{eff}} \frac{ch\nu_m dn_\mathrm{c}}{V_\mathrm{eff} n_g} \cdot \frac{\nu_{m'}}{dn_\mathrm{c}} \\ &= \frac{2cn_2 h\nu_m \nu_{m'}}{n_g n_\mathrm{eff} V_\mathrm{eff}}\end{aligned} \tag{2}$$

where we use $d\nu_{m',\mathrm{Kerr}}/\nu_{m'} = 2n_2 dI/n_\mathrm{eff}$. c is the speed of light, h is the Planck constant, $n_g = 2.1$ is the group index, $n_\mathrm{eff} = 1.8$ is the effective refractive index, V_eff is the effective optical mode volume, $n_2 = 2.4 \times 10^{-19}\mathrm{m}^2/\mathrm{W}$ is the nonlinear index of Si_3N_4. The factor of 2 comes from the cross-phase-modulation, as the pump and probe modes are two distinct resonances in our experiment (i.e. $m \neq m'$ and $\nu_m \neq \nu_{m'}$).

The thermal response term $\chi_\mathrm{therm}(0)$ is calculated as

$$\begin{aligned}\chi_\mathrm{therm}(0) &= \frac{d\nu_{m',\mathrm{therm}}}{dn_\mathrm{c}} \\ &= \frac{\nu_{m'}}{n_\mathrm{mat}} \cdot \frac{dn_\mathrm{mat}}{dn_\mathrm{c}} \\ &= \frac{\nu_{m'}}{n_\mathrm{mat}} \cdot \frac{dT}{dn_\mathrm{c}} \cdot \frac{dn_\mathrm{mat}}{dT} \\ &= \frac{\kappa_\mathrm{abs} h\nu_m \nu_{m'}}{n_\mathrm{mat}} \frac{dT}{dP_\mathrm{abs}} \frac{dn_\mathrm{mat}}{dT}\end{aligned} \tag{3}$$

where we use $d\nu_{m',\mathrm{therm}}/\nu_{m'} = dn_\mathrm{mat}/n_\mathrm{mat}$ and $dP_\mathrm{abs} = \kappa_\mathrm{abs} h\nu_m dn_\mathrm{c}$. The material refractive index of Si_3N_4 at 1550 nm is $n_\mathrm{mat} = 2.0$, and its thermo-optic coefficient[22] is $dn_\mathrm{mat}/dT = 2.5 \times 10^{-5}/\mathrm{K}$.

Supplementary Note 12. Fitting of the measured response

For the microresonator response data presented in Fig. 5 in the main manuscript, we use the fitting function below

$$\chi(\omega) = \chi_\mathrm{Kerr}(0) \cdot \left(1 + \frac{\chi_\mathrm{therm}(0)}{\chi_\mathrm{Kerr}(0)} \frac{\chi_\mathrm{therm}(\omega)}{\chi_\mathrm{therm}(0)}\right) \frac{1}{1+2i\omega/\kappa_\mathrm{probe}} \frac{1}{1+2i\omega/\kappa_\mathrm{pump}} \tag{4}$$

to extract the response ratio $\gamma = \frac{\chi_\mathrm{therm}(0)}{\chi_\mathrm{Kerr}(0)}$ from the response measurement. Here the free fitting parameters are only κ_pump, the ratio γ, and an arbitrary constant pre-factor. The normalized thermal response $\frac{\chi_\mathrm{therm}(\omega)}{\chi_\mathrm{therm}(0)}$ is retrieved from the frequency domain heat transfer COMSOL simulations, and κ_probe is measured and kept the same for all measurements performed on the same microresonator. Importantly, only the data above 10 kHz is used in the fitting due to the locking distortion at lower frequencies. The validity of the dynamical heating simulations, and hence that of the simulated response function, is verified by benchmarking the model with our recent thermorefractive noise measurement[23] of similar Si_3N_4 samples, where the measured noise spectrum is connected to the real part of our

response function through the Fluctuation-Dissipation Theorem (FDT) in the frequency range that we are interested in.

We notice that the fitting function (which we refer to as "analogue" fitting)

$$\chi'(\omega) = \chi_{\text{Kerr}}(0)\cdot\left(1+\frac{\chi_{\text{therm}}(0)}{\chi_{\text{Kerr}}(0)}\frac{1}{1+(\omega/\omega_{\text{th}})^{\zeta}}\right)\frac{1}{1+2i\omega/\kappa_{\text{probe}}}\frac{1}{1+2i\omega/\kappa_{\text{pump}}}, \tag{5}$$

where the "analogue" function $\frac{1}{1+(\omega/\omega_{\text{th}})^{\zeta}}$ with free parameters, thermal cutoff frequency ω_{th} and pole number ζ, replaces the simulated thermal response function $\frac{\chi_{\text{therm}}(\omega)}{\chi_{\text{therm}}(0)}$, could in principle fit the curve better to the measured response data. However, we observed that using this model, the fitting tends to systematically *overestimate* the response ratio γ for high absorption resonances as illustrated in Supplementary Fig. 11. This fitting artifact is manifested in the absorption rate calibration as shown in Supplementary Fig. 11(c), where for resonances with higher than 10 MHz absorption rate, the method starts to overestimate the absorption rate, yielding absorption rates that are *larger* than the actual, i.e. physically observed, absorption rates. This overestimate occurs due to the complicated thermal

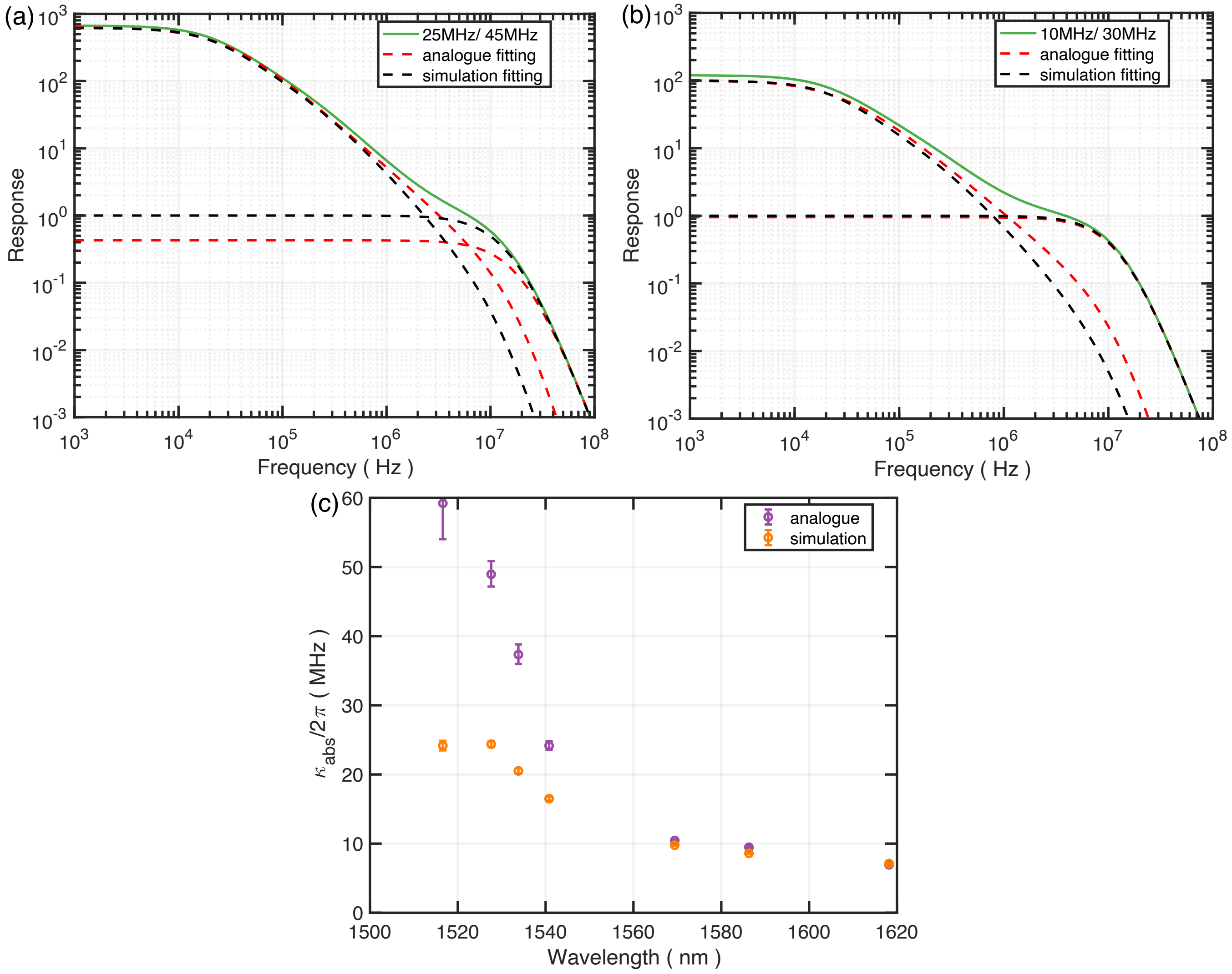

Supplementary Figure 11: Comparison of fitting results using the simulated thermal response versus a generic ("analogue") thermal response model. The fitting contributions from both the thermal effect and Kerr effect are shown, with the black dotted curves corresponding to the fitting result using fitting function $\chi(\omega)$ from Supplementary Eq. 4, and the red dotted curves corresponding to the fitting result using the analogue fitting function $\chi'(\omega)$ from Supplementary Eq. 5. Panel (a) is the simulated response function with absorption rate 25 MHz and cavity linewidth 45 MHz. Panel (b) is the simulated response function with absorption rate 10 MHz and cavity linewidth 30 MHz. One could easily see that the analogue fitting result does not correctly capture the thermal response of the device, and therefore tends to overestimate the response ratio γ for high absorption resonances leading to unphysically high intrinsic absorption that exceeds the measured cavity loss rate. This feature of the analogue fitting is reflected in the absorption rate calibration, as is shown in Panel (c), that for resonances with absorption rate higher than 10MHz the analogue fitting method starts to overestimate the absorption rate.

response of the cavity, which cannot be fully captured by a one-pole fitting model. The estimated absorption rate is therefore highly sensitive to the spectral dependence of the thermal response. To illustrate this point, we employ Bayesian Statistical Analysis[32]—a powerful statistical framework to analyse the posterior probability distribution of the fitting parameters (which can also take into account prior information). The correlations of the free fitting parameters of the analogue fitting function is shown in Supplementary Fig. 12 using the Markov Chain Monte Carlo method[31]. One can easily see that most parameters heavily correlates with ζ, especially the response ratio γ and the cavity linewidth $\kappa_{\mathrm{pump}}/2\pi$. The correlation is stronger for higher absorption resonances, and since ζ is estimated poorly using the analogue fitting method, the fitting will yield a poorly estimated (overestimated in our case) response ratio. Therefore, for responses where the Kerr contribution is not significant at the cavity cutoff frequency, only the fitting with the simulated thermal response function is recommended, as one could see in Supplementary Fig. 13 that this method has fewer fitting parameters and give physical estimation of both the response ratio γ and the cavity linewidth $\kappa_{\mathrm{pump}}/2\pi$.

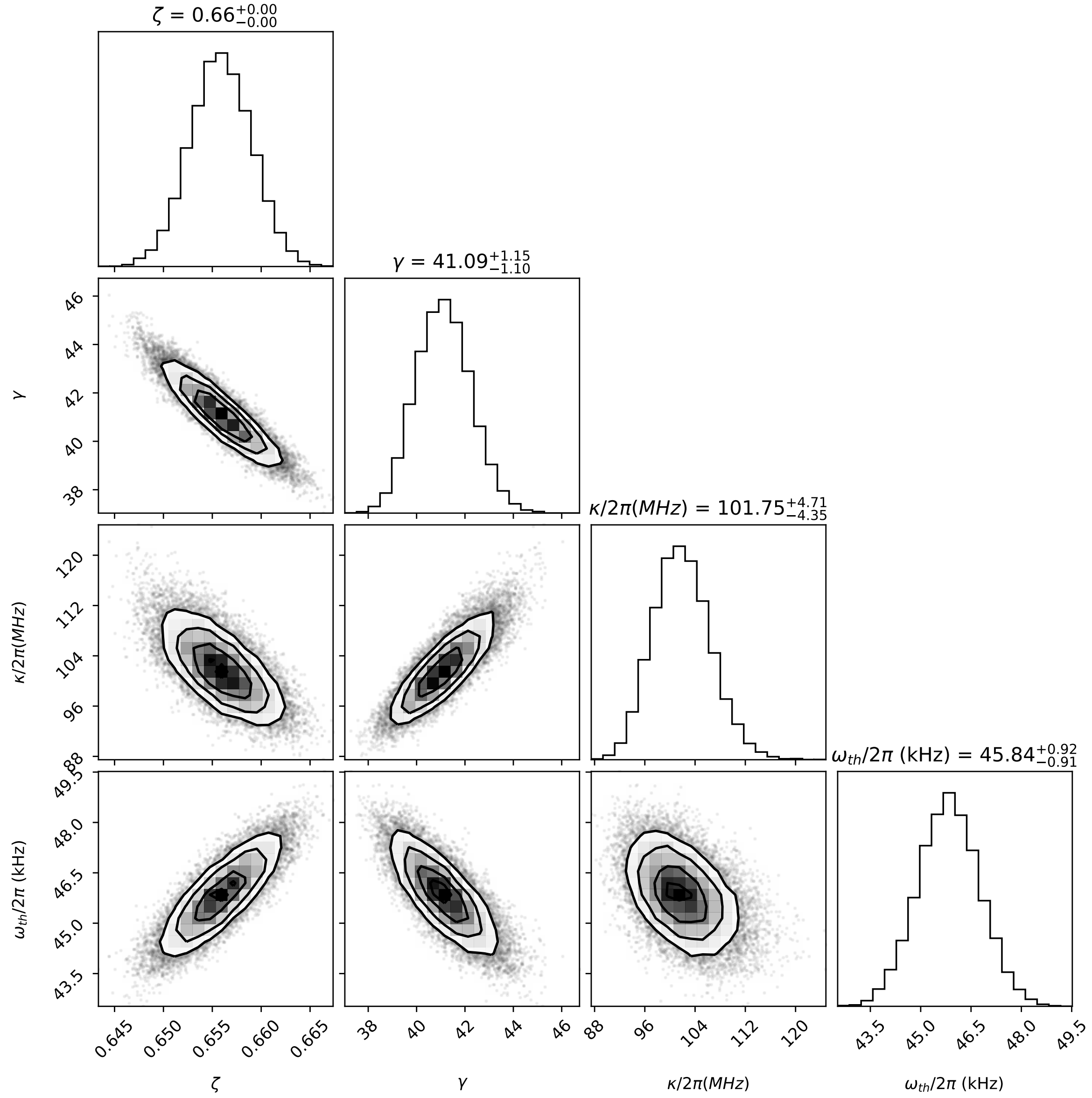

Supplementary Figure 12: Bayesian statistical analysis showing the posterior distribution of the parameters of the analogue fitting function and their correlations, using the Markov Chain Monte Carlo method. One can see that all the parameters heavily correlate to ζ, which is poorly estimated by the analogue fitting. This leads to an overestimation of the response ratio γ and the cavity linewidth $\kappa_{\mathrm{pump}}/2\pi$.

Extra response fitting data similar to that shown in Fig. 5(c, e) in the main manuscript are presented in Supplementary Fig. 14.

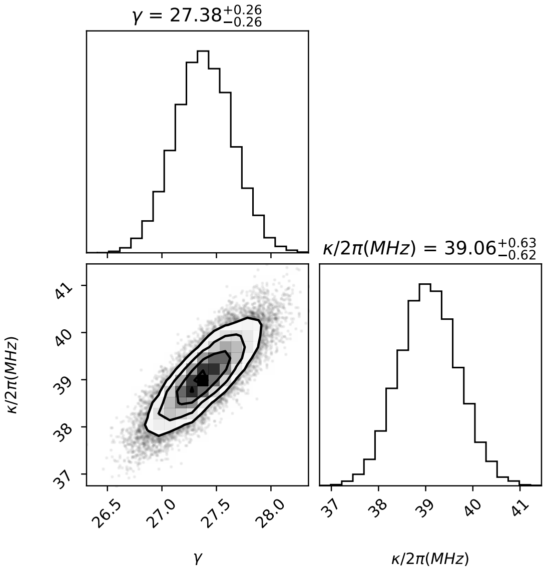

Supplementary Figure 13: Bayesian statistical analysis showing the posterior distribution of the parameters of the fitting function from Supplementary Eq. 4 and their correlation, using the Markov Chain Monte Carlo method. This fitting method leads to physical estimation of the response ratio γ and the cavity linewidth $\kappa_{\mathrm{pump}}/2\pi$.

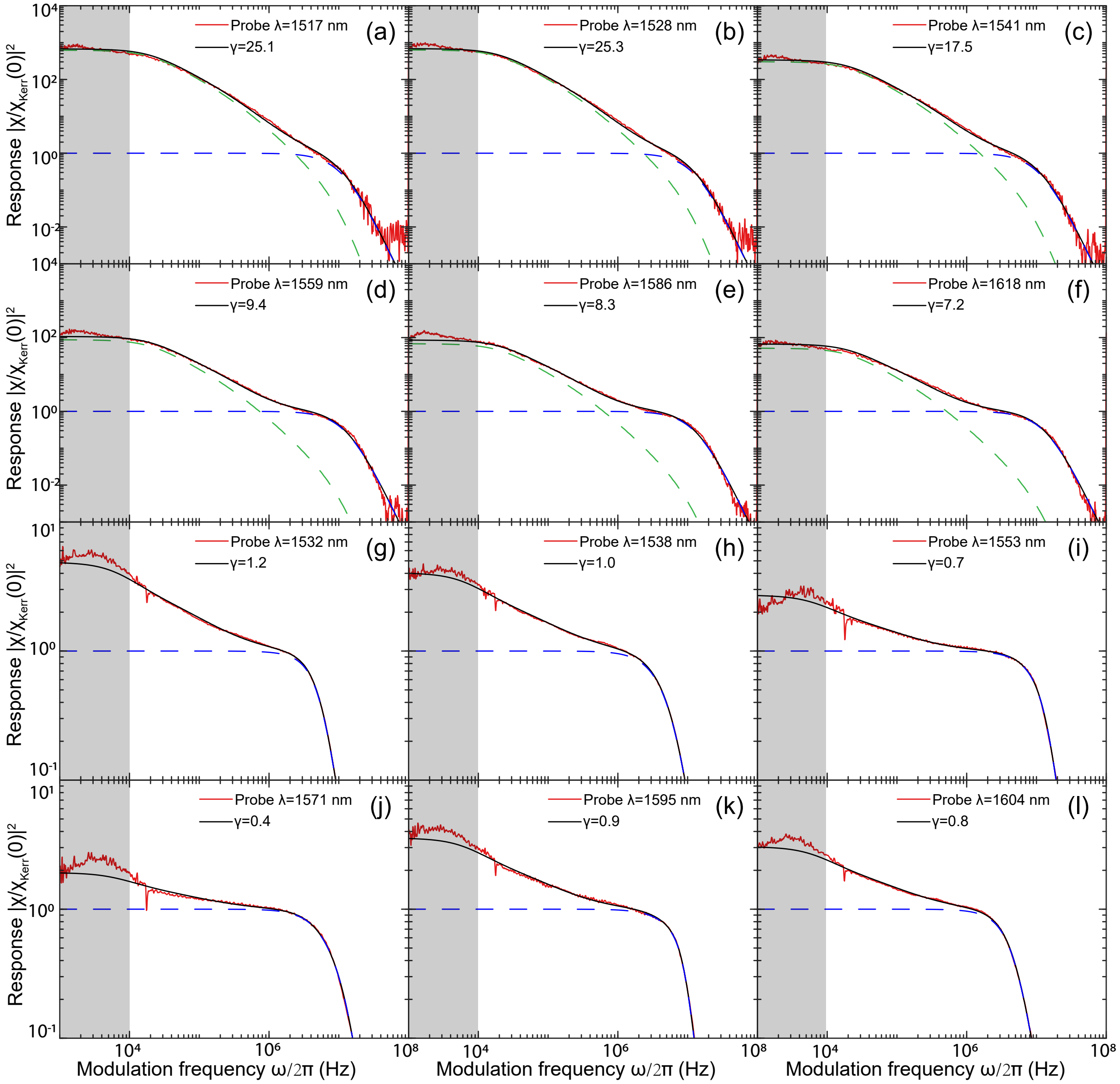

Supplementary Figure 14: More resonance response data fittings similar to those shown in Fig. 5(c, e) in the main manuscript. Panels (a – f) correspond to data shown in Fig. 5(c) in the main manuscript. Panels (g – l) correspond to 40-GHz-FSR data shown in Fig. 5(e) in the main manuscript.

Supplementary References

[1] Liu, J. *et al.* Ultralow-power chip-based soliton microcombs for photonic integration. *Optica* **5**, 1347–1353 (2018).
[2] Riemensberger, J. *et al.* Massively parallel coherent laser ranging using a soliton microcomb. *Nature* **581**, 164–170 (2020).
[3] Raja, A. S. *et al.* Chip-based soliton microcomb module using a hybrid semiconductor laser. *Opt. Express* **28**, 2714–2721 (2020).
[4] Hu, J. *et al.* Reconfigurable radiofrequency filters based on versatile soliton microcombs. *Nature Communications* **11**, 4377 (2020).
[5] Liu, J. *et al.* Photonic microwave generation in the X- and K-band using integrated soliton microcombs. *Nature Photonics* **14**, 486–491 (2020).
[6] Liu, J. *et al.* Monolithic piezoelectric control of soliton microcombs. *Nature* **583**, 385–390 (2020).
[7] Del'Haye, P., Arcizet, O., Gorodetsky, M. L., Holzwarth, R. & Kippenberg, T. J. Frequency comb assisted diode laser spectroscopy for measurement of microcavity dispersion. *Nature Photonics* **3**, 529–533 (2009).
[8] Liu, J. *et al.* Frequency-comb-assisted broadband precision spectroscopy with cascaded diode lasers. *Opt. Lett.* **41**, 3134–3137 (2016).
[9] Gorodetsky, M. L., Pryamikov, A. D. & Ilchenko, V. S. Rayleigh scattering in high-Q microspheres. *J. Opt. Soc. Am. B* **17**, 1051–1057 (2000).
[10] Li, Q., Eftekhar, A. A., Xia, Z. & Adibi, A. Unified approach to mode splitting and scattering loss in high-Q whispering-gallery-mode microresonators. *Phys. Rev. A* **88**, 033816 (2013).
[11] Herr, T. *et al.* Temporal solitons in optical microresonators. *Nature Photonics* **8**, 145 (2013).
[12] Guo, H. *et al.* Universal dynamics and deterministic switching of dissipative Kerr solitons in optical microresonators. *Nature Physics* **13**, 94 (2016).
[13] Pfeiffer, M. H. P. *et al.* Ultra-smooth silicon nitride waveguides based on the Damascene reflow process: fabrication and loss origins. *Optica* **5**, 884–892 (2018).
[14] Pfeiffer, M. H. P. *et al.* Octave-spanning dissipative Kerr soliton frequency combs in Si_3N_4 microresonators. *Optica* **4**, 684–691 (2017).
[15] Tian, H. *et al.* Hybrid integrated photonics using bulk acoustic resonators. *Nature Communications* **11**, 3073 (2020).
[16] Churaev, M. *et al.* Hybrid Si_3N_4-$LiNbO_3$ integrated platform for electro-optic conversion. In *Conference on Lasers and Electro-Optics*, STh1F.3 (Optical Society of America, 2020).
[17] Tikan, A. *et al.* Emergent nonlinear phenomena in a driven dissipative photonic dimer. *Preprint at http://arxiv.org/abs/2005.06470* (2020).
[18] Pfeiffer, M. H. P. *et al.* Photonic Damascene process for low-loss, high-confinement silicon nitride waveguides. *IEEE Journal of Selected Topics in Quantum Electronics* **24**, 1–11 (2018).
[19] Guo, H. *et al.* Mid-infrared frequency comb via coherent dispersive wave generation in silicon nitride nanophotonic waveguides. *Nat. Photonics* **12**, 330–335 (2018).
[20] Pfeiffer, M. H. P., Liu, J., Geiselmann, M. & Kippenberg, T. J. Coupling ideality of integrated planar high-Q microresonators. *Phys. Rev. Applied* **7**, 024026 (2017).
[21] Wilson, D. J. *et al.* Integrated gallium phosphide nonlinear photonics. *Nature Photonics* **14**, 57–62 (2020).
[22] Arbabi, A. & Goddard, L. L. Measurements of the refractive indices and thermo-optic coefficients of Si_3N_4 and SiO_x using microring resonances. *Opt. Lett.* **38**, 3878–3881 (2013).
[23] Huang, G. *et al.* Thermorefractive noise in silicon-nitride microresonators. *Physical Review A* **99**, 061801 (2019).
[24] Xuan, Y. *et al.* High-Q silicon nitride microresonators exhibiting low-power frequency comb initiation. *Optica* **3**, 1171–1180 (2016).
[25] Ji, X. *et al.* Ultra-low-loss on-chip resonators with sub-milliwatt parametric oscillation threshold. *Optica* **4**, 619–624 (2017).
[26] Ye, Z., Twayana, K., Andrekson, P. A. & Torres-Company, V. High-Q Si_3N_4 microresonators based on a subtractive processing for Kerr nonlinear optics. *Opt. Express* **27**, 35719–35727 (2019).
[27] Chiles, J. *et al.* Deuterated silicon nitride photonic devices for broadband optical frequency comb generation. *Opt. Lett.* **43**, 1527–1530 (2018).
[28] Dirani, H. E. *et al.* Ultralow-loss tightly confining Si_3N_4 waveguides and high-Q microresonators. *Opt. Express* **27**, 30726–30740 (2019).
[29] Wu, K. & Poon, A. W. Stress-released Si_3N_4 fabrication process for dispersion-engineered integrated silicon photonics. *Opt. Express* **28**, 17708–17722 (2020).
[30] Spencer, D. T., Bauters, J. F., Heck, M. J. R. & Bowers, J. E. Integrated waveguide coupled Si_3N_4 resonators in the ultrahigh-Q regime. *Optica* **1**, 153–157 (2014).
[31] Foreman-Mackey, D., Hogg, D. W., Lang, D. & Goodman, J. emcee: The MCMC Hammer. *PASP* **125**, 306-312 (2013).
[32] Gregory, Phil Bayesian logical data analysis for the physical sciences: a comparative approach with mathematica® support. 2005. Cambridge University Press
